# Circulating Polyamines and Metabolic Changes Following a Mediterranean Diet with or Without Naltrexone/Bupropion in Breast Cancer Survivors: An Exploratory Secondary Analysis

**DOI:** 10.3390/nu18101621

**Published:** 2026-05-20

**Authors:** Won-Jun Choi, Yu Ra Lee, Yae-Ji Lee, Yu-Jin Kwon, A-Ra Cho, Jeongae Lee, Ji Won Lee

**Affiliations:** 1 Department of Medicine, Graduate School, Yonsei University, Seoul 03722, Republic of Korea; slashwj@gmail.com; 2 Food Functionality Research Division, Korea Food Research Institute, Wanju-gun 55365, Republic of Korea; lyr@kfri.re.kr; 3 Department of Biostatistics and Computing, Yonsei University, Seoul 03722, Republic of Korea; ysbiostat@yuhs.ac; 4 Department of Family Medicine, Yonsei University College of Medicine, Severance Hospital, Seoul 03722, Republic of Korea; digda3@yuhs.ac; 5 Department of Family Medicine, Yonsei University College of Medicine, Yong-in Severance Hospital, Yong-in 16995, Republic of Korea; ara1713@yuhs.ac; 6 Center for Advanced Molecular Recognition, Korea Institute of Science and Technology, Seoul 02792, Republic of Korea

**Keywords:** breast cancer, Mediterranean diet, naltrexone/bupropion, polyamines, spermine, biomarker, *N*-acetylspermine

## Abstract

**Background/Objectives:** The Mediterranean diet is widely recognized for its cardiovascular and metabolic benefits, including weight reduction; however, the metabolic mechanisms underlying these effects remain incompletely understood. This study investigated whether changes in circulating polyamines are associated with metabolic improvements following a Mediterranean diet intervention, particularly in breast cancer survivors. **Methods:** This exploratory secondary analysis used stored paired serum samples from a previously reported 8-week controlled intervention conducted in three groups: Group A (breast cancer survivors following a Mediterranean diet alone, *n* = 21), Group B (breast cancer survivors following a Mediterranean diet combined with naltrexone/bupropion, *n* = 23), and Group C (non-cancer participants receiving the combined intervention, *n* = 28). Paired polyamine data were available for 16, 9, and 16 participants, respectively. Breast cancer survivors were randomized to Groups A and B, whereas Group C was enrolled as a non-randomized active comparison group. Serum metabolic profiles were analyzed using liquid chromatography–mass spectrometry-based untargeted metabolomics, and nine polyamines were quantified using targeted analysis. An exploratory indirect-effect analysis examined associations between changes in serum polyamines and clinical outcomes, including body composition and lipid parameters. **Results:** Body weight, fat mass, and Homeostatic Model Assessment of Insulin Resistance (HOMA-IR) decreased significantly within all three groups after the 8-week intervention (median changes: −1.9 to −2.8 kg, −1.9 to −2.8 kg, and −0.3 to −0.7, respectively). LDL cholesterol decreased significantly only within the two groups receiving naltrexone/bupropion (median changes: −20.6 and −10.1 mg/dL). However, between-group differences in these changes were not statistically significant. *N*-acetylspermine increased nominally in all groups (*p* < 0.01), whereas spermine increased only in the Mediterranean diet alone group (*p* = 0.015). **Conclusions:** Mediterranean diet-related metabolic improvements were accompanied by changes in circulating polyamines. Spermine and *N*-acetylspermine may represent candidate metabolic response markers associated with nutritional and pharmacological interventions in breast cancer survivorship.

## 1. Introduction

Breast cancer is one of the most common hormone-related malignancies, including estrogen receptor-positive and progesterone receptor-positive subtypes. According to the Ministry of Data and Statistics of the Republic of Korea, the overall incidence of cancer has been steadily increasing, with cancer-related deaths accounting for 27.0% of all deaths in 2020 [1]. Furthermore, data from the Korea Central Cancer Registry indicates that in 2022, breast cancer had the highest incidence among all cancers in women, with 111.6 cases per 100,000 individuals, and this rate has steadily increased over the past two decades [2].

The risk factors for breast cancer can be broadly categorized into genetic and environmental factors. Genetic predispositions, such as mutations in the *BRCA1/2* genes, are well-established, whereas environmental factors include diet, obesity, physical inactivity, and lifestyle habits [3,4,5]. Among these, the role of obesity remains a subject of ongoing debate; however, it has increasingly been recognized as a distinct risk factor for the development, recurrence, and metastasis of breast cancer [6,7].

Various strategies exist to manage obesity, including lifestyle interventions such as dietary modification and physical activity, with pharmacological treatment as an adjunct when necessary. If weight control can reduce the risk of breast cancer recurrence, dietary modification may serve as a primary intervention strategy.

The Mediterranean diet is regarded as one of the healthiest dietary patterns worldwide. It is characterized by a high intake of vegetables, fruits, whole grains, legumes, fish, and olive oil, which are typical of regions bordering the Mediterranean Sea. This diet has anti-inflammatory effects, reduces oxidative stress, and inhibits the expression of carcinogenic growth factors [8,9]. Moreover, nutrient-rich components such as beta-carotene, vitamins A and C, and dietary fiber are associated with a reduced risk of breast cancer [10].

In addition to dietary control, pharmacotherapy for obesity may improve patient outcomes. Contrave, a combination of low-dose naltrexone (8 mg) and bupropion (90 mg), has been approved for long-term weight management. Bupropion stimulates pro-opiomelanocortin (POMC) neurons in the hypothalamus, which play a key role in appetite suppression and energy expenditure. These neurons secrete neuropeptides such as α-melanocyte-stimulating hormone (α-MSH), which act on melanocortin receptors to suppress appetite. Bupropion has been shown in animal models to enhance POMC neuronal firing and increase α-MSH release, although the exact mechanisms remain incompletely understood [11].

Naltrexone, a μ-opioid receptor antagonist, augments the effect of bupropion by inhibiting autoinhibitory feedback on POMC neurons. Low-dose naltrexone has also been investigated in relation to opioid growth factor signaling and tumor-cell proliferation, although its relevance to breast cancer survivorship weight management remains incompletely established [12,13].

Polyamines are aliphatic compounds that bind directly to DNA, RNA, and ATP, and play essential roles in cell growth, cell differentiation, and gene regulation [14]. In addition to their roles in cellular proliferation, emerging evidence indicates that polyamines participate in metabolic regulation and energy homeostasis. Studies have shown that enhanced polyamine catabolism can prevent diet-induced obesity and improve glucose tolerance, suggesting a potential link between polyamine metabolism and obesity resistance [15,16]. Recent evidence further indicates that polyamines, particularly spermidine, play a role in metabolic regulation through modulation of mitochondrial function, autophagy, and insulin sensitivity [17]. Elevated polyamine levels have been observed in various cancer types, including breast cancer, and may serve as potential biomarkers for cancer screening, progression, and treatment response. Notably, increased levels of acetylated polyamines have been reported in breast tissue and serum from patients with breast cancer, supporting their potential utility as metabolic or clinical biomarkers rather than direct indicators of tumor progression [18,19,20].

The main clinical outcomes of the parent intervention have been reported previously [20], demonstrating that a Mediterranean diet, with or without naltrexone/bupropion, was associated with weight loss and metabolic improvements, without clear superiority of the combined intervention.

The present study extends these findings by examining previously unreported targeted polyamine profiling and untargeted serum metabolomics using stored paired samples. Although dietary patterns are known to influence circulating metabolomic profiles [21], metabolic response markers linking such interventions to clinical outcomes remain incompletely characterized, particularly in human studies, and evidence linking dietary interventions to circulating polyamine dynamics is limited.

Given prior experimental evidence linking polyamine metabolism to obesity resistance and glucose/lipid metabolism [18,19], we hypothesized that changes in circulating polyamines may be associated with metabolic responses to the intervention. Accordingly, this study investigated whether changes in circulating polyamines, particularly spermine and *N*-acetylspermine, were associated with changes in body composition and metabolic parameters following the intervention.

## 2. Materials and Methods

### 2.1. Chemicals and Materials

All chemicals used in this study were purchased from Sigma-Aldrich (St. Louis, MO, USA). All solvents were purchased from Burdick & Jackson (Muskegon, MI, USA). Water was filtered using a Millipore Milli-Q purification system (Bedford, MA, USA).

### 2.2. Study Design and Participants

The present study was an exploratory secondary analysis of stored serum samples obtained from a previously reported 8-week controlled intervention conducted at Gangnam Severance Hospital (Seoul, Republic of Korea) between July 2017 and July 2018. The original primary outcome of the parent trial was defined as changes in obesity-, inflammation-, and metabolism-related clinical indicators after an 8-week Mediterranean diet intervention with or without naltrexone/bupropion. Safety and tolerability monitoring, quality of life, sexual function, fatigue, and biospecimen collection for subsequent metabolomic analyses were prespecified as secondary or ancillary components. Ethical approval was granted by the Institutional Review Board of Gangnam Severance Hospital (IRB No. 3-2017-0097), and the study adhered to the ethical principles outlined in the Declaration of Helsinki. All participants provided written informed consent prior to enrollment. The trial was retrospectively registered on ClinicalTrials.gov (Identifier: NCT03581630, 6 July 2018).

The design and main clinical outcomes of the parent trial have been reported previously [20]. In brief, breast cancer survivors were randomly assigned to either a Mediterranean diet alone group or a Mediterranean diet plus naltrexone/bupropion group using a computer-generated random sequence with a block size of four. The randomization sequence was generated by an independent statistician, and participants were assigned to interventions by a study coordinator in the order of enrollment. No stratification factors were prespecified. Outcome assessors were blinded to group allocation; however, participants and intervention providers were not blinded due to the nature of the dietary and pharmacological interventions. Age-matched non-cancer participants were enrolled separately as a non-randomized active comparison group and received the Mediterranean diet combined with naltrexone/bupropion. Therefore, randomization was applied only to the two breast cancer survivor groups, and comparisons involving non-cancer participants should be interpreted as exploratory comparisons with a non-randomized group rather than as fully randomized between-group contrasts.

For the present analysis, participants were classified into three intervention groups: Group A (breast cancer survivors following a Mediterranean diet alone, *n* = 21), Group B (breast cancer survivors following a Mediterranean diet combined with naltrexone/bupropion, *n* = 23), and Group C (non-cancer participants following a Mediterranean diet combined with naltrexone/bupropion, *n* = 28). Breast cancer survivors and age-matched non-cancer participants were recruited through posters and online advertisements at Gangnam Severance Hospital. All volunteers were initially screened by telephone.

Women aged 20–65 years were eligible for inclusion if they had a body mass index (BMI) > 25.0 kg/m^2^ (obese for Asian populations, according to the World Health Organization) or a BMI > 23.0 kg/m^2^ (overweight for Asian populations) in the presence of at least one metabolic risk factor (waist circumference ≥ 80 cm, triglyceride levels ≥ 150 mg/dL, high-density lipoprotein (HDL) cholesterol levels < 50 mg/dL, fasting glucose levels ≥ 100 mg/dL, blood pressure ≥ 130/85 mmHg, or a diagnosis of hypertension, type 2 diabetes mellitus, or dyslipidemia currently controlled with medications). Participants diagnosed with stage I–III breast cancer who had completed cancer treatment, including surgery, adjuvant chemotherapy, radiotherapy, and/or hormonal therapy, were included in the breast cancer survivor group.

Participants who met any of the following criteria were excluded: uncontrolled hypertension; hepatic or renal disease; significant cardiovascular disease or stroke; history of seizures; serious psychiatric illness (such as bipolar disorder, schizophrenia, bulimia, anorexia nervosa, or suicidal ideation); use of medications such as monoamine oxidase inhibitors, opioid-containing medications, other naltrexone- or bupropion-containing medications, or tamoxifen; history of alcohol abuse or dependence; current smokers or use of nicotine replacement products within the previous 6 months; or women who were pregnant or breastfeeding. Participants with a history of breast cancer were excluded if they had experienced cancer recurrence or metastasis. Women of childbearing age were required to use effective contraception throughout the study period and for up to 30 days after discontinuation of the study drug.

**Figure 1:**
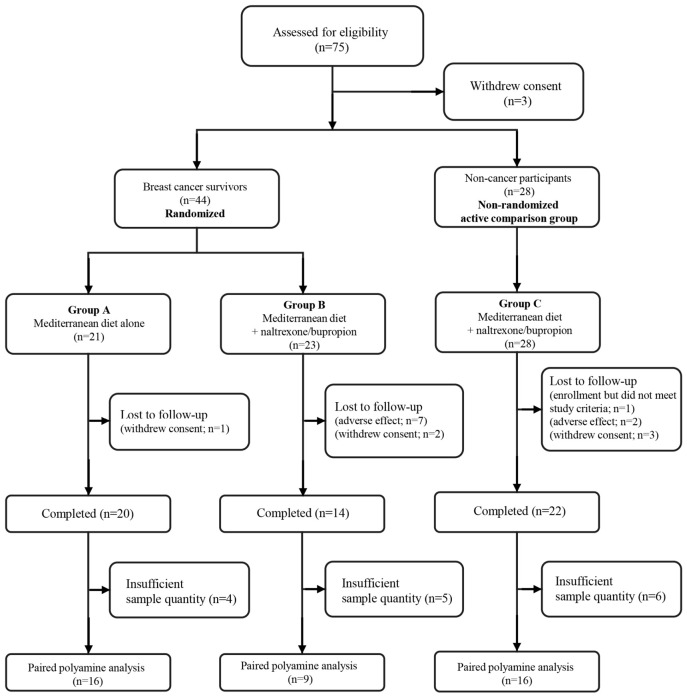
Flowchart of participant recruitment and sample selection for the present exploratory metabolomics analysis. The parent intervention included 21 breast cancer survivors receiving the Mediterranean diet alone, 23 breast cancer survivors receiving the Mediterranean diet plus naltrexone/bupropion, and 28 non-cancer participants receiving the combined intervention. Participants without paired baseline and week 8 serum samples or with insufficient targeted polyamine detection were excluded from the complete-case paired polyamine analysis. The final analytical sample included 16, 9, and 16 participants in Groups A, B, and C, respectively.

For the present paired polyamine analysis, participants were included only when paired baseline and week 8 serum samples were available, and targeted polyamine detection was sufficient for quantification. Participants without paired serum specimens or those with sample-availability or technical limitations that precluded reliable polyamine quantification were excluded from the complete-case metabolomics analysis. The final paired analytical sample included 16 participants in Group A, 9 in Group B, and 16 in Group C (Figure 1). Because this complete-case subset was smaller than the parent trial population, particularly in Group B, all metabolomic and indirect-effect analyses were considered exploratory.

### 2.3. Intervention and Adherence Assessment

The intervention protocol was based on a previously published randomized controlled trial [20], and only key elements relevant to the present analysis are summarized here.

At study initiation, participants received individualized guidance from trained nutrition professionals on adopting a Mediterranean-style dietary pattern. To support adherence, standardized meal examples reflecting Mediterranean diet principles were provided at multiple time points during the intervention.

Dietary adherence was evaluated using a modified 13-item Mediterranean Diet Adherence Screener derived from the PREDIMED instrument, with the alcohol-related item removed due to safety considerations in breast cancer survivors. Scores ranged from 0 to 13, and a threshold of ≥9 was used to indicate high adherence.

Participants were advised to emphasize plant-based foods and unsaturated fat sources, including vegetables, fruits, legumes, fish, nuts, and vegetable oils, while reducing consumption of red and processed meats, saturated fats, and refined sugar products. Energy intake targets were set at less than 1500 kcal per day.

Adherence monitoring incorporated multiple complementary approaches. Participants submitted daily meal images and self-reported adherence scores via a mobile-based platform, and the study team provided individualized feedback. In addition, dietary intake was assessed at baseline and week 8 using 24 h dietary recall interviews to estimate energy and nutrient intake.

Because a comprehensive Korean food-based polyamine database was not available, direct estimation of dietary polyamine intake was not feasible. Therefore, dietary exposure relevant to polyamine metabolism was inferred indirectly using adherence scores, dietary recall data, and food group-based information from the adherence screener.

Participants assigned to the pharmacological intervention received an extended-release combination of naltrexone (8 mg) and bupropion (90 mg). Treatment was initiated at 1 tablet daily and increased to 2 tablets daily, administered in divided doses, if tolerated. Medication adherence was assessed by pill counts at scheduled visits, and participants with adherence below 80% were excluded from further analysis.

### 2.4. Sample Collection and Outcome Measurements

Participants’ body weights (measured to the nearest 0.1 kg) were recorded while wearing light clothing. Height was measured (to the nearest 0.1 cm) using an automatic extensometer (BSM 330; Biospace, Seoul, Republic of Korea). BMI was calculated by dividing weight (kg) by the square of height (m^2^). Body composition was assessed using a bioelectrical impedance analyzer (ACCUNIQ BC720; SELVAS Healthcare Inc., Daejeon, Republic of Korea) to measure skeletal muscle mass, fat mass, and fat percentage. Waist circumference was measured at the midpoint between the lowest rib and the iliac crest with the participants in a standing position.

Fasting blood samples were collected from the antecubital vein at baseline and after the 8-week intervention to assess metabolic parameters. White blood cell (WBC) counts were measured using an XN-9000 hematology analyzer (Sysmex America Inc., Lincolnshire, IL, USA). The ADVIA 1650 Clinical Chemistry System (Siemens Medical Solutions, Tarrytown, NY, USA) was used to determine fasting glucose, high-sensitivity *C*-reactive protein, total cholesterol, triglyceride levels, and HDL cholesterol levels. When triglyceride levels were ≤400 mg/dL, low-density lipoprotein (LDL) cholesterol levels were calculated using the Friedewald equation as follows: LDL cholesterol = total cholesterol − HDL cholesterol − triglycerides/5, with all lipid values expressed in mg/dL. Fasting insulin levels were measured using an electrochemiluminescence immunoassay with the Elecsys 2010 analyzer (Roche, Indianapolis, IN, USA). Insulin resistance was calculated using the Homeostatic Model Assessment of Insulin Resistance (HOMA-IR) formula: HOMA-IR = (fasting insulin [μIU/mL] × fasting glucose [mg/dL])/405.

Participants were evaluated at baseline, week 1, week 4, and week 8, with interim assessments for adverse events and medication adherence. Safety evaluations included monitoring treatment-emergent adverse events, concomitant medications, vital signs (recorded at each visit), and clinical laboratory measurements (recorded at baseline and at week 8), including serum creatinine levels and liver function tests. At each visit, adverse events were recorded and categorized by severity (mild, moderate, or severe) and by potential relatedness to the intervention, as assessed by the study investigators.

### 2.5. Untargeted Metabolite Profiling

Serum samples for untargeted metabolite profiling were prepared in 100 μL aliquots, and 400 μL of acetonitrile was added for protein precipitation. This was followed by centrifugation using Ultrafree^®^-MC-VV centrifugal filters (MilliporeSigma, Burlington, MA, USA) at 1200× *g* for 5 min.

The liquid chromatography-mass spectrometry (LC-MS) conditions applied were the same as those described previously [22]. Metabolic profiling was performed using an ACQUITY™ UPLC system (Waters, Milford, MA, USA) coupled to a Q-Tof Premier™ quadrupole/time-of-flight hybrid mass spectrometer (Waters, Milford, MA, USA). Chromatographic separation was conducted using an ACQUITY UPLC BEH C18 column (2.1 × 100 mm, 1.7 μm; Waters, Milford, MA, USA) at a flow rate of 0.35 mL/min. The gradient elution system comprised solvent A (water with 0.1% formic acid) and solvent B (acetonitrile with 0.1% formic acid), and was programmed as follows: 0–3 min, 5% B; 3–10 min, 5–50% B; 10–11.5 min, 50–95% B; and 11.5–12 min, 95–5% B. The gradient was then returned to the initial concentration (5% B) and maintained for 2 min before the next sample was analyzed. The column and autosampler temperatures were maintained at 40 °C and 4 °C, respectively. The injection volume was 5 μL. The mass spectrometer was operated in both positive and negative ionization modes for accurate mass measurements. Samples were analyzed in full-scan mode with an *m*/*z* range of 50–1200 and a mass window of 0.05 Da. The data were processed using MassLynx 4.1 software (Waters, Milford, MA, USA).

### 2.6. Targeted Metabolite Profiling of Polyamines

For targeted metabolite profiling, 800 μL of acetonitrile was added to 200 μL of each serum sample for protein precipitation, followed by the addition of an internal standard, 1,6-diaminohexane (1 µg/mL × 20 μL). After protein precipitation, the samples were centrifuged at 1200× *g* for 5 min using a Smart R17 Plus centrifuge (Hanil, Kimpo, Republic of Korea). The supernatants were transferred to a 10 mL tube, to which 100 μL of dansyl chloride (4 mg/mL in acetonitrile) and 100 μL of sodium carbonate buffer (0.1 M, pH 9.0) were added. The mixture was incubated at 60 °C for 15 min. After evaporation, the residue was reconstituted with 100 μL of methanol.

LC separation was performed using a Shiseido Nanospace SI-2 HPLC system (Osaka Soda, Osaka, Japan) coupled with an LTQ XL ion trap MS (Thermo Fisher Scientific, Waltham, MA, USA). Eluent A consisted of water with 0.1% formic acid in 5% acetonitrile, and eluent B consisted of acetonitrile with 0.1% formic acid in 5% water. Samples were analyzed using a Hypersil GOLD C18 column (150 × 2.1 mm inner diameter; 3 μm; Thermo Fisher, Waltham, MA, USA) under the following gradient conditions: 0 min, 12% B; 0–17 min, 12–88% B (held for 8 min); and 25–28 min, 88–12% B, at a flow rate of 0.2 mL/min and a temperature of 35 °C. The injection volume was 5 μL. The mass spectrometer was operated in positive electrospray ionization mode. Raw data were collected and processed using Xcalibur software version 4.1 (Thermo Fisher Scientific, Waltham, MA, USA).

### 2.7. Statistical Analysis

Retention time and mass-to-charge (*m*/*z*) ratio were evaluated using MassLynx 4.1 software. Raw LC–MS data were processed using MassLynx 4.1 software, including baseline correction, scaling, peak alignment, and matrix manipulation. The MarkerLynx application manager within MassLynx was used to detect ions of interest from the MS dataset and to construct the data matrix based on chromatographic peaks, *m*/*z* values, retention times, and ion intensities.

The feature matrix generated from MarkerLynx was uploaded to MetaboAnalyst 6.0 for multivariate analysis. Feature filtering was performed using the interquartile range (IQR) criterion to remove variables with low variability prior to multivariate analysis. After data filtering and integrity checking, no missing values were detected in the uploaded matrix; therefore, no missing-value imputation was performed. The retained data were log-transformed and Pareto-scaled prior to multivariate analysis to reduce heteroscedasticity and improve comparability across metabolites.

Partial least squares discriminant analysis (PLS-DA) was performed using MetaboAnalyst 6.0 to visualize pre- and post-intervention differences in untargeted serum metabolomic profiles within each study group and ionization mode. After the above preprocessing procedures, all retained metabolomic features were included in the PLS-DA models; no supervised feature selection was performed before model construction.

Model performance was assessed using R^2^ and Q^2^ values, representing explained variance and cross-validated predictive ability, respectively. The number of latent components and Q^2^ values were determined using 5-fold cross-validation in MetaboAnalyst 6.0. To evaluate model robustness and potential overfitting, permutation testing was performed for each PLS-DA model using 100 random permutations of class labels while keeping the metabolomic data matrix unchanged. Empirical *p* values were calculated by comparing the classification accuracy of the original PLS-DA model with the distribution of classification accuracies obtained from the permuted models.

Variable importance in projection (VIP) scores were used only for post hoc interpretation of variables contributing to class separation and were not used to preselect features before PLS-DA modeling. For heatmap visualization, the top 20% of metabolomic features were selected based on *t*-test *p* value ranking between pre- and post-intervention samples. Heatmaps were generated to visualize relative differences in feature intensities before and after the intervention. These feature-ranking procedures were considered exploratory and were not used as independent validation of the PLS-DA models.

Continuous variables were summarized as medians with interquartile ranges because of the small sample size and non-normal distributions. Clinical variables, including body composition, serum metabolic parameters, and vital signs, were compared before and after the 8-week intervention within each group using the Wilcoxon signed-rank test. Between-group differences at baseline were evaluated using the Kruskal–Wallis test. Between-group differences in changes from baseline were evaluated using the Kruskal–Wallis test or rank-based analysis of covariance adjusted for age and baseline BMI, as appropriate.

For targeted polyamine quantification, changes in serum concentrations before and after the intervention were analyzed within groups using non-parametric paired tests, and comparisons across groups were performed using non-parametric between-group tests. Because this was an exploratory secondary analysis with a small complete-case sample and multiple targeted polyamine tests, no formal multiplicity correction was applied. All *p* values from targeted polyamine analyses were reported as nominal and interpreted as hypothesis-generating.

Exploratory indirect-effect analyses were performed to evaluate whether changes in serum polyamines were statistically associated with changes in body composition or serum metabolic parameters. These analyses used the R ‘mediation’ package and generated average causal mediation effect (ACME), average direct effect (ADE), total effect, and proportion-mediated estimates with 95% confidence intervals based on nonparametric bootstrap resampling. ACME terminology follows the output of the R mediation package; however, because the present analysis was exploratory, based on a small complete-case sample, and included a non-randomized active comparison group, ACME estimates were interpreted as exploratory indirect-effect estimates rather than evidence of causal mediation. These models were adjusted for age and baseline BMI. Analyses were conducted for the overall cohort and for the naltrexone/bupropion-treated subgroup comprising Groups B and C. Nominal *p* values are presented for indirect-effect analyses, and results were interpreted cautiously as hypothesis-generating.

Although intention-to-treat and per-protocol analyses were prespecified in the parent trial, the present metabolomics analysis was restricted to participants with paired baseline and week 8 serum samples and sufficient polyamine detection.

## 3. Results

### 3.1. Analytical Sample and Baseline Characteristics

Of the 72 participants in the parent intervention, 41 had paired baseline and week 8 serum samples with sufficient targeted polyamine detection and were included in the present complete-case metabolomics analysis: 16 in Group A, 9 in Group B, and 16 in Group C (Figure 1). Table 1 summarizes baseline values and changes from baseline in this analytical sample. At baseline, the groups were generally similar in age, height, body-composition measures, and most serum metabolic parameters, whereas systolic blood pressure (SBP) and heart rate (HR) differed among groups. Because Group C was a non-randomized active comparison group and the complete-case sample was small, baseline characteristics and between-group comparisons were interpreted descriptively. The full dataset, including post-intervention values and inter-group comparisons for post-intervention and change values, is provided in Table S1.

### 3.2. Intervention Adherence and Dietary Intake

Adherence to the Mediterranean diet improved in the parent intervention cohort [20]. The proportion of participants with high adherence, defined as a modified Mediterranean diet score ≥ 9, increased from 37.5% at baseline to 85.7% at week 8 overall. By group, the proportion increased from 40.0% to 90.0% in Group A, from 42.9% to 92.9% in Group B, and from 31.8% to 77.3% in Group C. In the parent-trial dietary assessment, median total energy intake decreased from 1653.8 to 1386.2 kcal/day (*p* = 0.001), the MUFA/SFA ratio increased from 1.24 to 1.35 (*p* = 0.046), and dietary fiber intake increased from 13.5 to 18.2 g/1000 kcal (*p* < 0.001). Physical activity, assessed using the GLTEQ score, also increased after the intervention (*p* < 0.001). Between-group differences in nutrient intake and physical activity changes were not significant in the parent trial. Direct dietary polyamine intake was not quantified in the present analysis.

### 3.3. Changes in Body Composition, Serum Metabolic Parameters, and Vital Signs

In within-group analyses, body weight, BMI, waist circumference, fat mass, fat percentage, and HOMA-IR decreased significantly across all three groups (all *p* < 0.05), whereas skeletal muscle mass did not change significantly. WHR showed small but statistically significant changes in all groups (Table 1).

Among metabolic parameters, fasting glucose decreased in Groups A and C but not in Group B, while insulin decreased significantly only in Group B. QUICKI increased in Groups A and C but not in Group B, and WBC count decreased only in Group A.

**Table 1:** Baseline Values and Changes in Body Composition, Metabolic Parameters and Vital Signs Following the 8-Week Intervention.

Characteristic	Breast Cancer Survivors						Non-Cancer Participants			Baseline
	A Group (MeDiet Alone)			B Group (MeDiet + NB)			C Group (MeDiet + NB)			
	(*n* = 16)			(*n* = 9)			(*n* = 16)			
	Baseline	Change	*p*-Value	Baseline	Change	*p*-Value	Baseline	Change	*p*-Value	
Age	55.0 (52.8, 57.5)			56.0 (56.0, 57.0)			55.0 (53.0, 57.5)			0.531
Height (cm)	158.7 (154.8, 161.2)			157.0 (155.6, 161.5)			156.2 (153.5, 159.2)			0.366
Body Composition										
Weight (kg)	66.3 (61.8, 68.8)	−1.9 (−3.2, −1.2)	<0.001 *	66.5 (63.2, 68.0)	−2.8 (−3.9, −2.3)	0.009 *	66.5 (63.0, 73.2)	−2.3 (−3.0, −1.5)	<0.001 *	0.658
BMI (kg/m^2^)	25.6 (24.9, 26.7)	−0.8 (−1.3, −0.5)	<0.001 *	27.0 (24.6, 27.6)	−1.1 (−1.6, −0.9)	0.004 *	28.3 (25.4, 30.0)	−1.0 (−1.2, −0.6)	<0.001 *	0.273
WC (cm)	88.5 (85.8, 93.0)	−5.8 (−7.3, −2.4)	0.005 *	93.4 (88.0, 94.0)	−5.0 (−7.7, −4.4)	0.013 *	92.5 (90.1, 97.4)	−3.3 (−5.9, −0.8)	0.017 *	0.187
Skeletal muscle (kg)	21.9 (21.3, 22.6)	0.0 (−0.7, 0.6)	0.851	22.3 (21.0, 22.9)	0.1 (−0.3, 0.3)	1.000	21.8 (20.8, 23.2)	0.0 (−0.2, 0.1)	0.726	0.906
Fat mass (kg)	25.3 (20.6, 29.5)	−1.9 (−2.4, −1.0)	<0.001 *	24.2 (22.9, 28.6)	−2.8 (−3.7, −1.8)	0.004 *	27.8 (21.8, 31.7)	−2.3 (−2.9, −0.7)	0.010 *	0.618
Fat percentage	38.3 (35.2, 42.7)	−1.8 (−2.9, −0.6)	0.006 *	38.5 (36.2, 42.4)	−2.5 (−4.0, −1.6)	0.004 *	40.0 (34.9, 43.7)	−1.9 (−3.0, −1.1)	0.006 *	0.677
WHR	0.9 (0.9, 1.0)	0.0 (0.0, 0.0)	0.014 *	1.0 (0.9, 1.0)	0.0 (−0.1, 0.0)	0.014 *	1.0 (0.9, 1.0)	0.0 (0.0, 0.0)	0.009 *	0.549
Metabolic Parameters										
WBC (no./μL)	5.2 (4.7, 5.6)	−0.8 (−1.0, −0.2)	0.013 *	5.6 (5.0, 6.1)	−0.1 (−0.7, 0.7)	0.820	5.3 (4.8, 6.0)	0.2 (−0.4, 1.1)	0.389	0.535
Fasting glucose (mg/dL)	84.5 (79.0, 92.3)	−11.0 (−13.3, −3.5)	0.004 *	85.0 (82.0, 90.0)	−10.0 (−17.0, −4.0)	0.059	88.0 (82.0, 97.0)	−12.0 (−14.3, −6.8)	<0.001 *	0.652
Insulin (μIU/mL)	7.0 (5.2, 8.7)	−0.9 (−1.6, −0.1)	0.073	7.5 (5.3, 11.6)	−2.6 (−4.1, −0.7)	0.039 *	6.3 (5.1, 9.8)	−1.1 (−2.1, 0.2)	0.088	0.779
HOMA-IR	1.4 (1.1, 2.0)	−0.4 (−0.5, −0.1)	0.034 *	1.5 (1.0, 2.7)	−0.7 (−1.1, −0.3)	0.027 *	1.5 (1.1, 2.3)	−0.3 (−0.7, −0.1)	0.013 *	0.791
QUICKI	0.4 (0.3, 0.4)	0.0 (0.0, 0.0)	0.008 *	0.4 (0.3, 0.4)	0.0 (0.0, 0.0)	0.055	0.4 (0.3, 0.4)	0.0 (0.0, 0.0)	0.025 *	0.791
Total Cholesterol (mg/dL)	202.0 (182.8, 222.8)	−16.0 (−24.0, 4.5)	0.093	179.0 (152.0, 197.0)	−27.0 (−37.8, −19.3)	0.022 *	191.5 (157.8, 212.8)	−17.0 (−21.5, −1.5)	0.020 *	0.226
Triglyceride (mg/dL)	127.0 (102.8, 178.5)	−26.0 (−51.0, 3.0)	0.021 *	123.0 (121.0, 150.0)	−38.0 (−44.5, −9.0)	0.161	100.0 (81.8, 143.3)	−2.5 (−18.8, 23.5)	0.959	0.172
HDL cholesterol (mg/dL)	54.0 (48.5, 65.1)	−1.7 (−2.6, 3.3)	1.000	56.6 (50.6, 60.0)	−0.6 (−2.8, −0.1)	0.183	64.5 (54.2, 71.1)	−1.4 (−5.7, 3.6)	0.744	0.125
LDL cholesterol (mg/dL)	113.5 (99.9, 137.3)	−7.7 (−18.1, 11.6)	0.518	109.9 (63.8, 120.7)	−20.6 (−27.0, −11.9)	0.039 *	116.9 (79.4, 128.6)	−10.1 (−29.4, −0.3)	0.008 *	0.499
Vital Signs										
SBP	129.0 (124.3, 138.3)	−8.5 (−19.8, −3.5)	0.012 *	121.0 (117.0, 122.0)	−4.0 (−9.0, 6.0)	0.678	125.5 (122.8, 132.8)	−3.5 (−12.3, 0.5)	0.103	0.035 *
DBP	87.0 (77.8, 95.8)	−4.0 (−12.5, 3.0)	0.155	78.0 (77.0, 81.0)	−2.0 (−5.0, 1.0)	0.400	82.5 (77.0, 87.3)	−3.0 (−4.3, 0.3)	0.040 *	0.171
HR	78.0 (73.5, 81.8)	−6.5 (−9.3, −1.3)	0.019 *	68.0 (65.0, 74.0)	1.0 (0.0, 4.0)	0.175	76.0 (73.0, 85.0)	1.5 (−5.0, 8.8)	0.469	0.048 *

Data are presented as medians (interquartile ranges). * *p*  <  0.05 vs. baseline values within each group by Wilcoxon’s signed-rank test. Abbreviations: BMI, body mass index; WC, waist circumference; WHR, waist-to-hip ratio; WBC, white blood cell; HOMA-IR, homeostasis model assessment of insulin resistance; QUICKI, quantitative insulin sensitivity check index; HDL, high-density lipoprotein; LDL, low-density lipoprotein; SBP, systolic blood pressure; DBP, diastolic blood pressure; HR, heart rate; MeDiet, Mediterranean diet; NB, naltrexone/bupropion. The rightmost *p* value refers to the between-group comparison of baseline values. Post-intervention values and between-group comparisons of post-intervention and change values are presented in Table S1.

For lipid profiles, total cholesterol and LDL cholesterol decreased in Groups B and C but not in Group A, whereas triglycerides decreased only in Group A. HDL cholesterol did not change significantly in any group.

Regarding vital signs, systolic blood pressure and heart rate decreased in Group A, whereas diastolic blood pressure decreased in Group C, with no significant changes observed in the other groups.

Between-group differences in changes were generally not statistically significant. In light of the small analytical sample and the non-randomized inclusion of Group C, the results are best interpreted as descriptive pre- and post-changes within each group rather than evidence of comparative superiority between interventions.

### 3.4. Safety and Tolerability

Safety outcomes are summarized from the parent intervention trial [20] as the present analysis was limited to a subset of participants with available paired metabolomic samples and was not designed to evaluate safety endpoints.

In the parent trial, a total of 51 participants were exposed to naltrexone/bupropion, of whom 39 (76%) reported at least one adverse event. The most commonly reported symptoms were gastrointestinal or neurologic in nature, including nausea (39%), dizziness (33%), dry mouth (25%), and constipation (22%). The majority of these events were classified as mild to moderate in severity.

A single serious adverse event (syncope) was reported in a non-cancer participant and was judged unlikely to be related to the study medication. Treatment discontinuation due to adverse events occurred in nine participants, most frequently because of nausea (10%), followed by urticaria (4%), dizziness (2%), and syncope (2%).

Given that safety was not specifically assessed in the metabolomics subset, these findings should be interpreted as reflecting the overall study population rather than the analytical sample included in the present analysis.

### 3.5. Serum Metabolomic Profile Changes in Breast Cancer Survivors and Non-Cancer Participants Following Intervention

**Figure 2:**
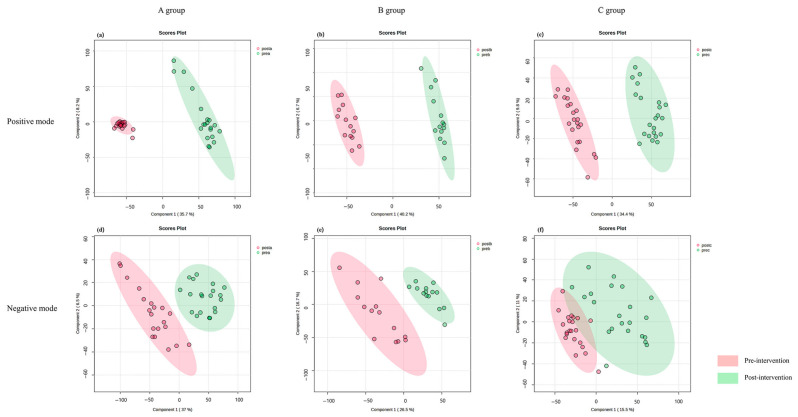
Partial least squares discriminant analysis (PLS-DA) score plots showing metabolomic profile differences before and after the intervention. (**a**–**c**) Positive mode; (**d**–**f**) negative mode. (**a**,**d**) Group A (breast cancer survivors, Mediterranean diet alone); (**b**,**e**) Group B (breast cancer survivors, Mediterranean diet and naltrexone/bupropion); (**c**,**f**) Group C (non-cancer participants, Mediterranean diet and naltrexone/bupropion). Permutation testing was performed for each PLS-DA model using 100 random permutations of class labels. Five of the six models showed empirical *p* values < 0.01, whereas the Group B positive-ion model did not reach statistical significance (empirical *p* = 0.07). Therefore, the Group B positive-ion separation should be interpreted cautiously.

To explore changes in serum metabolomic profiles before and after the 8-week intervention, PLS-DA was performed separately for each study group and ionization mode. The resulting score plots are presented in Figure 2. The PLS-DA plots suggested apparent separation between pre- and post-intervention samples in all groups, indicating possible intervention-associated shifts in serum metabolomic profiles. In the positive ion mode, separation patterns were observed in Groups A, B, and C (Figure 2a–c). In the negative ion mode, corresponding patterns are shown in Figure 2d–f.

Model performance was assessed using R^2^ and Q^2^ values, representing explained variance and predictive ability, respectively. After preprocessing, 567 metabolomic features in the positive ion mode and 26 features in the negative ion mode were included in the PLS-DA analyses. In the positive ion mode, the models showed high R^2^ and Q^2^ values (R^2^ = 0.94–0.98; Q^2^ = 0.92–0.96; Figure 2a–c). In contrast, the negative ion mode showed comparatively lower predictive performance (R^2^ = 0.63–0.74; Q^2^ = 0.36–0.69; Figure 2d–f). To further evaluate model robustness and potential overfitting, permutation testing was performed for each PLS-DA model using 100 random permutations of class labels while keeping the metabolomic data matrix unchanged. Five of the six PLS-DA models showed statistically significant permutation results, with empirical *p* values < 0.01. However, the Group B positive-ion model did not reach statistical significance in permutation testing (empirical *p* = 0.07). Therefore, although apparent separation was observed in this model, it should be interpreted cautiously as exploratory rather than as confirmatory evidence of robust discrimination. Overall, the PLS-DA findings suggest possible intervention-associated metabolomic shifts, but these results should be considered exploratory given the small sample size and the possibility of model instability.

**Figure 3:**
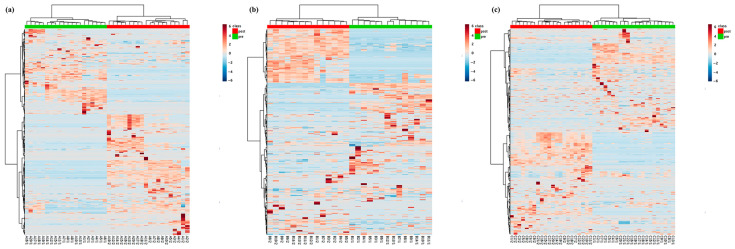
Heatmap showing the top 20% differential metabolites in the positive ion mode for each study group. (**a**) Group A (breast cancer survivors, Mediterranean diet alone); (**b**) Group B (breast cancer survivors, Mediterranean diet and naltrexone/bupropion); (**c**) Group C (non-cancer participants, Mediterranean diet and naltrexone/bupropion).

To further visualize the metabolic changes, heatmap analyses were performed using MetaboAnalyst 6.0 to represent the top 20% of metabolomic features ranked by *t*-test *p* values in the positive ion mode for each group, as shown in Figure 3. These heatmaps distinguished between upregulated and downregulated metabolites before and after the intervention and showed clustering patterns consistent with the PLS-DA results. The heatmaps provided additional descriptive visualization of pre- and post-clustering patterns and were not used as independent validation of the PLS-DA models.

### 3.6. Quantification of Polyamines in Serum Samples from Breast Cancer Survivors and Non-Cancer Participants

Table 2 presents baseline levels and changes in nine serum polyamines following the 8-week intervention. Within-group paired analyses showed nominal increases in *N*-acetylspermine in all three groups: Group A, +12.5 (4.7, 35.0), *p* = 0.002; Group B, +28.6 (5.2, 37.8), *p* = 0.004; and Group C, +18.5 (4.9, 30.9), *p* = 0.002 (Table 2). This pattern suggests that *N*-acetylspermine may be a candidate polyamine response marker for further investigation. Several polyamines differed among groups at baseline, including *N*-acetylputrescine, 1,3-diaminopropane, putrescine, cadaverine, *N*-acetylspermidine, spermidine, and spermine (all baseline between-group *p* < 0.05; Table 2). Therefore, between-group comparisons of polyamine concentrations and changes were interpreted cautiously.

Spermine increased nominally only in Group A, with a median change of +52.7 (2.5, 196.1), *p* = 0.015, whereas no nominal within-group changes were observed in Group B, +150.5 (24.9, 230.1), *p* = 0.625, or Group C, +55.5 (11.7, 210.5), *p* = 0.074. This group-specific pattern may warrant further investigation into whether the Mediterranean diet alone influences circulating spermine levels in breast cancer survivors.

No other polyamines showed nominal within-group changes after the intervention in any group. The full dataset, including post-intervention concentrations and statistical comparisons among the three groups for post-intervention and change values, is provided in Table S2.

### 3.7. Exploratory Indirect-Effect Analyses of Serum Polyamines, Body Composition and Serum Metabolic Parameters

Exploratory indirect-effect analyses were performed to examine whether changes in serum polyamines were statistically associated with intervention-related changes in body composition and serum metabolic parameters. The intervention (8-week Mediterranean diet program with or without pharmacotherapy) was treated as the exposure, changes in serum polyamines from baseline were treated as candidate mediators, and corresponding changes in metabolic parameters were treated as outcomes. All models were adjusted for age and baseline BMI. Table 3 summarizes selected exploratory indirect-effect estimates for serum metabolic parameters; full results are provided in Table S3.

Across all participants, spermine showed a nominal indirect-effect estimate for total cholesterol, corresponding to the ACME output from the R mediation package (ACME = −4.82, 95% CI: −11.85 to −0.01; *p* = 0.046), with a nominal total effect (TE = −13.99, 95% CI: −27.15 to −0.96; *p* = 0.032). These findings suggest that changes in serum spermine may be associated with the observed reduction in total cholesterol; however, causal interpretation should be made cautiously. Full mediation results for all metabolic parameters are provided in Table S3.

In the overall cohort, no serum polyamine showed nominal indirect-effect estimates for body-composition outcomes (Table S4). This suggests that although certain polyamines were associated with changes in serum metabolic outcomes, statistically significant mediation effects for body composition were not consistently observed in this study.

Among patients who received naltrexone/bupropion treatment (Groups B and C), a subgroup mediation analysis was conducted to assess whether serum polyamines contributed to body composition changes following the intervention. Consistent with the primary analysis, changes in serum polyamines were tested as mediators of changes in body composition metrics within this specific pharmacologically treated population. Table 4 highlights the significant mediating effects on body composition. In the naltrexone/bupropion-treated subgroup, *N*-acetylspermine showed nominal indirect-effect estimates for weight (ACME = −1.66, 95% CI: −3.57 to −0.25; *p* = 0.020), waist circumference (ACME = −2.03, 95% CI: −4.66 to −0.04; *p* = 0.046), and fat mass (ACME = −1.04, 95% CI: −2.31 to −0.08; *p* = 0.030). These subgroup findings were considered exploratory and hypothesis-generating. The ACME was −1.66 for weight (*p* = 0.020), −2.03 for waist circumference (*p* = 0.046), and −1.04 for fat mass (*p* = 0.030), each indicating statistically significant average mediation effects. The total effects were also significant for waist circumference and fat mass, reinforcing the potential relevance of *N*-acetylspermine in body composition changes. The full dataset, including non-significant results, is provided in Table S5.

In contrast, no significant mediating effects of polyamines were observed on serum metabolic parameters (e.g., glucose, insulin, and lipid profiles) within the same treatment group (Table S6).

## 4. Discussion

In this exploratory secondary analysis of stored serum samples from a previously reported 8-week controlled intervention, we examined changes in circulating polyamines and their associations with body composition and serum metabolic parameters in breast cancer survivors and non-cancer participants. In the complete-case analytical sample, within-group paired analyses showed reductions in weight, BMI, waist circumference, fat mass, and HOMA-IR in all three groups. Given that Group C was not randomized, these findings should be interpreted as descriptive pre and post changes rather than evidence of comparative efficacy between interventions.

Exploratory indirect-effect analyses suggested nominal associations involving spermine with total cholesterol in the overall analytical sample and *N*-acetylspermine with weight, waist circumference, and fat mass in the naltrexone/bupropion-treated subgroup. Because these analyses were based on a small complete-case sample, used nominal *p* values without multiplicity adjustment, and included a non-randomized active comparison group, the findings should be interpreted as hypothesis-generating associations rather than evidence of causal mediation.

The parent trial previously reported that the Mediterranean diet, with or without naltrexone/bupropion, was associated with weight loss, improved metabolic parameters, and enhanced quality of life, while the combined intervention did not produce superior changes compared with the Mediterranean diet alone. In the present complete-case metabolomics analysis, the clinical variables were used primarily to contextualize circulating polyamine changes and exploratory indirect-effect estimates.

Intervention adherence provides important context for interpreting the polyamine findings. In the parent intervention cohort, high Mediterranean diet adherence increased substantially after the intervention, and dietary assessment showed reductions in total energy intake with increases in the MUFA/SFA ratio and dietary fiber intake [20]. However, direct dietary polyamine intake was not quantified in the present analysis. Therefore, the observed circulating polyamine changes cannot be attributed specifically to dietary polyamine exposure, and the dietary data should be interpreted as adherence and dietary-context indicators rather than direct measures of polyamine intake.

Beyond confirming the known benefits of the Mediterranean diet, this study examined the underexplored role of serum polyamines, particularly spermine and *N*-acetylspermine, as metabolic response markers associated with dietary and pharmacological interventions. Untargeted metabolomics analyses, including PLS-DA and heatmap analyses, suggested possible shifts in serum metabolomic profiles before and after the intervention. However, these findings should be interpreted cautiously because permutation testing did not uniformly support all PLS-DA models and because supervised metabolomic models can be unstable in small samples. In particular, the Group B positive-ion model did not reach statistical significance in permutation testing, indicating that the apparent separation in this model may be unstable and that overfitting cannot be excluded. Therefore, the untargeted metabolomics results are best interpreted as exploratory and hypothesis-generating. In contrast, targeted quantitative assays showed a nominal increase in *N*-acetylspermine levels in all groups and a nominal group-specific increase in spermine levels in the Mediterranean diet-alone group (Group A).

The observed increases in selected circulating polyamines are biologically plausible in light of prior reports that several Mediterranean diet components contain polyamines; however, this explanation remains indirect because dietary polyamine intake was not quantified in the present analysis. A global food supply analysis reported positive correlations between dietary spermine intake and typical Mediterranean components, including olive oil, seafood, fruits, cheese, and wine [23,24]. After ingestion, spermine is acetylated to *N*-acetylspermine by spermidine/spermine N1-acetyltransferase (SSAT), a key enzyme in polyamine catabolism that also regulates lipid and glucose metabolism [25,26,27].

The reason for the significant increase in *N*-acetylspermine levels across all three groups, in contrast to the increase in spermine levels observed only in the Mediterranean diet alone group, remains unclear. Although *N*-acetylspermine is traditionally considered a byproduct of spermine acetylation via SSAT, its elevation in Groups B and C, despite no nominal within-group increase in serum spermine, this pattern raises the possibility of additional regulatory mechanisms. SSAT activity can be influenced not only by intracellular spermine but also by hormones, cytokines, cellular stress, and pharmacological agents [16,27]. The pharmacological intervention in Groups B and C provides an additional context for interpretation; however, the present study did not measure SSAT activity or related molecular regulators, and therefore any mechanistic contribution of pharmacological treatment remains speculative. These findings underscore the need for further research on polyamine intake and SSAT regulation to clarify the underlying mechanisms [27,28].

The exploratory indirect-effect analysis provided hypothesis-generating evidence for an association involving spermine and total cholesterol. In the overall analytical sample, spermine showed a nominal indirect-effect estimate for total cholesterol. Experimental literature provides a biological context for this observation: spermine has been linked to the upregulation of peroxisome proliferator-activated receptor gamma coactivator-1α (PGC-1α) via SSAT-dependent modulation of the Akt pathway [28]. Activation of PGC-1α, in turn, enhances the expression of cholesterol 7 alpha-hydroxylase (CYP7A1), a key enzyme in bile acid synthesis, thereby promoting cholesterol catabolism and excretion [29,30]. However, these mechanistic pathways were not directly evaluated in the present clinical study and should therefore be considered biologically plausible interpretations requiring further investigation.

*N*-acetylspermine showed nominal indirect-effect estimates for weight, waist circumference, and fat mass in participants receiving naltrexone/bupropion. Because *N*-acetylspermine is commonly discussed in relation to intracellular spermine acetylation via SSAT, these findings raise the possibility that endogenous polyamine metabolism may be involved; however, the present study cannot disentangle endogenous regulation from dietary exposure or pharmacological context. Experimental studies have demonstrated that SSAT plays a crucial role in adiposity, with overexpression leading to reduced fat mass and knockout resulting in increased fat accumulation, potentially through the consumption of acetyl coenzyme A (acetyl-CoA) and suppression of lipogenesis [14]. Additionally, spermine may inhibit adipocyte differentiation by downregulating transcription factors such as CCAAT/enhancer-binding protein α and peroxisome proliferator-activated receptor γ [15]. Therefore, the observed increase in *N*-acetylspermine may altered polyamine catabolism, but whether this change is functionally linked to lipogenesis or lipid oxidation requires direct molecular validation.

The mediating effects observed in the naltrexone/bupropion group may partly reflect the additional metabolic effects of these agents. Although naltrexone/bupropion may influence energy balance through central mechanisms [10,27,29,31,32,33], the present analysis did not assess central signaling, SSAT activity, or mitochondrial pathways. Therefore, the subgroup indirect-effect findings should be viewed as exploratory signals requiring mechanistic confirmation. The subgroup findings in participants receiving naltrexone/bupropion may reflect the combined dietary and pharmacological context, but the study design does not allow separation of medication effects from cancer survivorship status or dietary exposure. This dual mechanism may accelerate *N*-acetylspermine production and promote lipid utilization, potentially via the acetyl-CoA consumption pathway. However, this proposed pathway remains to be confirmed through further experimental research.

Although within-group changes in insulin, fasting glucose, and WBC counts were observed after the intervention, no nominal indirect-effect estimates involving polyamines were observed for these outcomes in the relevant analyses. This suggests that they may result from mechanisms independent of polyamine metabolism or that the mediation analysis used was insufficiently sensitive to detect such effects. Further studies incorporating broader biomarker panels are needed to clarify the interplay between metabolic and immune pathways.

This study advances the understanding of polyamine metabolism—particularly spermine and *N*-acetylspermine—as candidate metabolic biomarkers associated with clinical responses, including changes in adiposity and lipid metabolism, rather than definitive mechanistic mediators. Integrating metabolomics and mediation analyses provides supportive evidence suggesting potential biological pathways underlying metabolic improvements, although further mechanistic validation remains warranted. From the perspective of breast cancer survivorship nutrition management, these findings have important clinical implications. Survivors often struggle with weight gain and metabolic dysregulation, which are known risk factors for recurrence [6,7]. These findings support further investigation of Mediterranean diet-based metabolic interventions, with or without pharmacological support, in breast cancer survivorship care. Circulating polyamines may be considered candidate metabolic response markers for future studies evaluating nutritional interventions. Collectively, these findings highlight the potential for an integrative approach to metabolic management in breast cancer survivors by leveraging changes in polyamine metabolism.

Because elevated polyamine levels have been reported in cancer-related contexts [16,18,19], increases in circulating polyamines should be interpreted cautiously, particularly in breast cancer survivors. In the present intervention setting, these changes occurred alongside metabolic improvements, but the study did not evaluate cancer recurrence, tumor activity, or long-term oncologic outcomes. In contrast, the present study suggests that increases in circulating polyamines associated with dietary intake through the Mediterranean diet may be linked to beneficial metabolic responses. This distinction from earlier biomarker-focused interpretations represents a potential strength of the present study.

Several limitations should be considered. First, the present analysis was an exploratory secondary analysis restricted to participants with paired serum samples and sufficient targeted polyamine detection. The complete-case sample was small, particularly in Group B, which limits statistical power and increases the risk of unstable or false-positive findings. Sensitivity analyses were not performed because the complete-case sample, particularly in Group B (*n* = 9), was too small to yield stable, reliable estimates from additional subgroup or exclusion analyses. Second, no formal multiplicity correction was applied for targeted polyamine or indirect-effect analyses; therefore, nominal *p* values should be interpreted as hypothesis-generating. Third, the study design did not include a non-cancer group receiving the Mediterranean diet alone, and Group C was enrolled as a non-randomized active comparison group. Therefore, the effects of diet, pharmacological treatment, cancer survivorship status, and their possible interactions cannot be separated. Importantly, this study should not be interpreted as a fully randomized three-arm comparative trial, but rather as a partially randomized design with an additional non-randomized comparison group. Fourth, direct dietary polyamine intake was not quantified; therefore, changes in circulating polyamines cannot be attributed specifically to exogenous dietary polyamine exposure or endogenous polyamine regulation. but rather should be interpreted as reflecting overall dietary pattern-related metabolic responses. Fifth, although cross-validation and permutation testing were used to assess PLS-DA model performance, one model did not reach statistical significance in permutation testing, and PLS-DA-based separation should be interpreted as exploratory rather than confirmatory pattern recognition, rather than evidence of robust group discrimination. Sixth, the parent trial was retrospectively registered, participants and intervention providers were not blinded, and only outcome assessors were blinded, which may have introduced performance bias and limited protocol transparency. Finally, the intervention duration was limited to 8 weeks, and the study was not designed to evaluate long-term weight maintenance, long-term safety, cancer recurrence, or survival outcomes.

## 5. Conclusions

This exploratory secondary analysis indicates that metabolic improvements following an 8-week Mediterranean diet intervention, with or without naltrexone/bupropion, were accompanied by changes in circulating polyamines. Exploratory indirect-effect analyses identified nominal associations of spermine with total cholesterol in the overall sample and of *N*-acetylspermine with body weight, waist circumference, and fat mass in the naltrexone/bupropion-treated subgroup. These findings support further investigation of circulating polyamines as candidate metabolic response markers in dietary and pharmacological interventions, particularly in breast cancer survivors.

## Figures and Tables

**Table 2:** Baseline Levels and Changes in Serum Polyamines Following the 8-Week Intervention.

	Breast Cancer Survivors						Non-Cancer Participants			Baseline
	A Group (MeDiet Alone)			B Group (MeDiet + NB)			C Group (MeDiet + NB)			
	(*n* = 16)			(*n* = 9)			(*n* = 16)			
	Baseline	Change	*p*-Value	Baseline	Change	*p*-Value	Baseline	Change	*p*-Value	
N_PUT	34.0 (28.5, 58.4)	−4.0 (−16.4, 1.8)	0.175	49.9 (36.3, 64.3)	13.0 (−12.0, 35.6)	0.250	69.0 (61.1, 91.3)	−3.1 (−19.5, 16.4)	0.970	0.001 *
N_CAD	9.3 (6.0, 14.8)	−1.0 (−8.0, 1.3)	0.414	13.5 (9.5, 17.5)	1.7 (−3.4, 6.6)	0.820	12.8 (9.5, 25.0)	−1.3 (−4.4, 4.6)	1.000	0.362
DAP	3.4 (3.0, 3.8)	0.4 (0.4, 0.4)	1.000	3.7 (3.0, 4.8)	0.5 (−0.1, 1.4)	0.625	5.3 (4.5, 6.4)	−0.4 (−1.3, 1.3)	0.846	0.018 *
PUT	29.7 (23.9, 50.5)	−1.0 (−14.1, 9.6)	0.782	34.1 (25.7, 43.8)	15.5 (−1.9, 27.7)	0.074	56.8 (49.9, 69.3)	−10.7 (−19.7, 24.9)	0.903	0.012 *
CAD	7.0 (4.3, 10.8)	5.8 (−7.9, 11.6)	0.638	13.8 (6.6, 28.4)	9.2 (0.5, 21.7)	0.563	19.9 (14.8, 31.8)	−8.9 (−10.6, 14.8)	0.813	0.046 *
N_SPD	49.0 (29.5, 72.8)	−2.8 (−22.7, 9.6)	0.274	58.4 (54.4, 91.3)	34.9 (−17.6, 56.2)	0.129	86.3 (78.5, 95.1)	−8.4 (−16.5, 57.9)	0.946	0.005 *
SPD	36.1 (24.4, 49.8)	3.1 (−8.2, 26.6)	0.231	119.1 (69.7, 138.5)	−2.0 (−33.2, 11.1)	0.820	77.2 (53.2, 129.7)	14.7 (−9.5, 32.4)	0.404	0.003 *
N_SPM	12.2 (6.2, 16.6)	12.5 (4.7, 35.0)	0.002 *	11.6 (9.0, 15.8)	28.6 (5.2, 37.8)	0.004 *	11.6 (8.0, 18.5)	18.5 (4.9, 30.9)	0.002 *	0.971
SPM	85.9 (59.3, 113.8)	52.7 (2.5, 196.1)	0.015 *	181.1 (130.2, 365.2)	150.5 (24.9, 230.1)	0.625	281.5 (202.7, 322.0)	55.5 (11.7, 210.5)	0.074	0.003 *

Data are presented as medians (interquartile ranges). * *p*  <  0.05 vs. baseline values within each group by Wilcoxon’s signed-rank test. *p* values for targeted polyamine analyses are nominal because no formal multiplicity correction was applied. Between-group comparisons were conducted using Quade’s rank analysis of covariance, adjusted for age and baseline BMI. Abbreviations: BMI, body mass index; MeDiet, Mediterranean diet; NB, naltrexone/bupropion; N_PUT, *N*-acetylputrescine; N_CAD, *N*-acetylcadaverine; DAP, 1,3-diaminopropane; PUT, putrescine; CAD, cadaverine; N_SPD, *N*-acetylspermidine; SPD, spermidine; N_SPM, *N*-acetylspermine; SPM, spermine. Post-intervention values and inter-group comparisons for post-intervention and change values are presented in Table S2.

**Table 3:** Selected Exploratory Indirect-Effect Estimates for Serum Polyamines and Serum Metabolic Parameters in All Participants.

		Total Cholesterol (mg/dL)		Triglyceride (mg/dL)		HDL Cholesterol (mg/dL)		LDL Cholesterol (mg/dL)	
		Estimate (95% CI)	*p*-Value	Estimate (95% CI)	*p*-Value	Estimate (95% CI)	*p*-Value	Estimate (95% CI)	*p*-Value
N_PUT	ACME	−0.67 (−4.55, 2.21)	0.700	1.60 (−7.02, 10.55)	0.716	0.01 (−0.94, 0.99)	0.970	−0.93 (−6.27, 3.74)	0.652
	ADE	−15.48 (−29.49, −1.78)	0.026 *	−22.90 (−44.32, −2.59)	0.032 *	0.73 (−5.79, 7.37)	0.854	−14.18 (−28.17, −0.61)	0.042 *
	Total Effect	−16.16 (−31.12, −2.38)	0.022 *	−21.29 (−43.95, 1.71)	0.066	0.74 (−5.87, 7.48)	0.848	−15.11 (−29.83, −0.23)	0.048 *
	Prop. Mediated	0.03 (−0.25, 0.33)	0.698	−0.04 (−1.37, 0.88)	0.770	0.00 (−1.03, 1.44)	0.914	0.05 (−0.49, 0.64)	0.644
N_CAD	ACME	−0.17 (−2.53, 2.26)	0.854	0.23 (−2.90, 3.81)	0.872	−0.05 (−1.29, 1.04)	0.950	−0.01 (−2.40, 2.44)	0.998
	ADE	−13.72 (−27.67, −0.15)	0.048 *	−18.10 (−42.15, 5.29)	0.124	1.14 (−6.06, 7.99)	0.746	−14.13 (−30.01, 0.89)	0.070
	Total Effect	−13.89 (−28.36, −0.11)	0.040 *	−17.87 (−42.59, 5.26)	0.124	1.08 (−6.14, 8.14)	0.760	−14.14 (−30.11, 0.96)	0.062
	Prop. Mediated	0.00 (−0.40, 0.36)	0.842	0.00 (−0.68, 0.39)	0.900	0.00 (−1.23, 1.75)	0.970	0.00 (−0.32, 0.34)	0.984
DAP	ACME	−0.18 (−4.27, 3.78)	0.944	−1.42 (−16.28, 10.99)	0.798	0.13 (−2.46, 3.44)	0.984	−0.05 (−4.49, 4.77)	0.968
	ADE	−13.42 (−31.56, 6.84)	0.182	−5.64 (−34.17, 24.21)	0.716	1.50 (−12.04, 16.59)	0.804	−14.33 (−36.64, 6.75)	0.180
	Total Effect	−13.59 (−31.90, 6.55)	0.190	−7.06 (−39.14, 22.38)	0.668	1.63 (−12.24, 16.72)	0.810	−14.38 (−36.66, 5.95)	0.196
	Prop. Mediated	0.01 (−0.55, 0.71)	0.914	0.10 (−5.50, 3.88)	0.770	0.01 (−1.67, 1.57)	0.866	0.00 (−0.98, 0.94)	0.908
PUT	ACME	−1.75 (−6.58, 2.24)	0.350	1.43 (−2.22, 7.58)	0.542	0.20 (−0.82, 1.58)	0.720	−2.22 (−8.51, 2.84)	0.368
	ADE	−13.76 (−26.87, −0.46)	0.038 *	−20.67 (−42.84, 2.76)	0.070	0.14 (−5.97, 6.16)	0.938	−13.00 (−25.99, 1.30)	0.074
	Total Effect	−15.51 (−28.49, −1.80)	0.026 *	−19.24 (−41.98, 3.62)	0.088	0.34 (−5.67, 6.47)	0.880	−15.22 (−29.12, 0.47)	0.058
	Prop. Mediated	0.10 (−0.30, 0.60)	0.348	−0.04 (−0.95, 0.30)	0.586	0.01 (−1.50, 1.66)	0.840	0.13 (−0.36, 0.74)	0.362
CAD	ACME	−2.15 (−10.26, 5.99)	0.536	0.73 (−4.43, 7.66)	0.834	−0.25 (−2.13, 1.03)	0.778	−2.10 (−11.00, 4.98)	0.564
	ADE	−10.58 (−28.51, 6.49)	0.228	−21.23 (−47.30, 5.25)	0.112	−2.33 (−9.03, 4.42)	0.480	−4.77 (−19.46, 10.22)	0.546
	Total Effect	−12.73 (−31.23, 5.41)	0.190	−20.49 (−46.13, 5.91)	0.124	−2.58 (−9.27, 4.44)	0.420	−6.87 (−24.87, 9.00)	0.412
	Prop. Mediated	0.15 (−2.01, 2.62)	0.546	−0.01 (−0.83, 0.95)	0.886	0.03 (−1.63, 1.91)	0.770	0.21 (−2.26, 3.68)	0.552
N_SPD	ACME	−1.85 (−6.87, 2.03)	0.348	1.61 (−2.18, 7.40)	0.458	−0.11 (−1.48, 1.00)	0.844	−1.83 (−7.19, 1.95)	0.348
	ADE	−13.56 (−27.48, 0.16)	0.056	−20.58 (−43.03, 1.30)	0.062	0.55 (−5.86, 6.85)	0.846	−13.05 (−27.16, 0.57)	0.070
	Total Effect	−15.41 (−30.64, −1.36)	0.030 *	−18.96 (−41.97, 2.62)	0.086	0.44 (−6.07, 6.70)	0.876	−14.88 (−29.46, −0.71)	0.042*
	Prop. Mediated	0.10 (−0.26, 0.75)	0.358	−0.05 (−1.19, 0.38)	0.500	0.00 (−1.96, 1.44)	0.988	0.10 (−0.27, 0.85)	0.358
SPD	ACME	−0.63 (−3.50, 1.32)	0.572	−1.53 (−7.86, 2.60)	0.510	0.05 (−1.03, 1.23)	0.932	−0.91 (−4.38, 1.63)	0.484
	ADE	−13.54 (−26.68, −0.56)	0.040 *	−15.02 (−37.27, 4.60)	0.136	0.61 (−5.65, 7.27)	0.866	−12.97 (−26.44, 1.38)	0.070
	Total Effect	−14.17 (−27.30, −1.10)	0.030 *	−16.55 (−37.73, 3.69)	0.122	0.66 (−5.64, 7.28)	0.854	−13.87 (−27.69, 0.31)	0.054
	Prop. Mediated	0.03 (−0.15, 0.40)	0.578	0.06 (−0.91, 0.95)	0.540	0.00 (−1.27, 1.72)	0.954	0.05 (−0.31, 0.50)	0.490
N_SPM	ACME	−6.31 (−13.98, 1.17)	0.100	2.50 (−8.97, 14.90)	0.656	−2.04 (−5.36, 0.74)	0.152	−3.32 (−12.60, 4.04)	0.402
	ADE	−6.15 (−21.12, 9.68)	0.440	−13.84 (−35.68, 9.43)	0.254	0.94 (−4.71, 7.06)	0.744	−8.86 (−24.95, 7.21)	0.278
	Total Effect	−12.45 (−26.40, 0.99)	0.082	−11.34 (−30.81, 9.36)	0.296	−1.10 (−6.57, 4.41)	0.658	−12.17 (−25.77, 1.96)	0.098
	Prop. Mediated	0.47 (−1.72, 3.22)	0.178	−0.13 (−3.73, 4.07)	0.772	0.40 (−10.91, 10.61)	0.702	0.22 (−1.33, 2.12)	0.464
SPM	ACME	−4.82 (−11.85, −0.01)	0.046 *	−0.86 (−10.67, 8.64)	0.838	−1.08 (−3.38, 0.60)	0.222	−2.56 (−9.09, 1.97)	0.262
	ADE	−9.17 (−22.06, 3.38)	0.164	−23.69 (−55.30, 5.82)	0.120	0.79 (−5.26, 6.87)	0.792	−10.20 (−25.28, 5.28)	0.184
	Total Effect	−13.99 (−27.15, −0.96)	0.032 *	−24.55 (−52.97, 4.03)	0.090	−0.30 (−6.21, 5.83)	0.928	−12.76 (−27.67, 1.94)	0.088
	Prop. Mediated	0.32 (−0.04, 1.52)	0.045 *	0.03 (−0.88, 1.38)	0.852	0.04 (−5.02, 7.81)	0.938	0.16 (−0.69, 1.71)	0.326

Data represent estimates from mediation analysis using nonparametric bootstrap (1000 iterations). ACME, ADE, and total effect estimates are presented with 95% confidence intervals (CI). * Nominal *p* < 0.05. *p* values are nominal and were not adjusted for multiplicity. ACME and ADE terminology follows the output of the R mediation package and should be interpreted as exploratory indirect-effect estimates rather than evidence of causal mediation. Analyses were adjusted for age and baseline BMI. Abbreviations: BMI, body mass index; HDL, high-density lipoprotein; LDL, low-density lipoprotein; N_PUT, *N*-acetylputrescine; N_CAD, *N*-acetylcadaverine; DAP, 1,3-diaminopropane; PUT, putrescine; CAD, cadaverine; N_SPD, *N*-acetylspermidine; SPD, spermidine; N_SPM, *N*-acetylspermine; SPM, spermine; ACME, average causal mediation effect; ADE, average direct effect; CI, confidence interval. Full exploratory indirect-effect estimates are presented in Table S3.

**Table 4:** Selected Exploratory Indirect-Effect Estimates for Serum Polyamines and Body Composition in Naltrexone/Bupropion-Treated Participants.

		Weight (kg)		WC (cm)		Fat Mass (kg)	
		Estimate (95% CI)	*p*-Value	Estimate (95% CI)	*p*-Value	Estimate (95% CI)	*p*-Value
N_PUT	ACME	−0.19 (−1.05, 0.39)	0.588	−0.17 (−1.11, 0.60)	0.660	0.12 (−0.24, 0.67)	0.584
	ADE	−2.54 (−5.12, −0.01)	0.048 *	−3.83 (−6.91, −0.94)	0.006 *	−2.26 (−3.99, −0.50)	0.014 *
	Total Effect	−2.73 (−5.38, −0.15)	0.042 *	−4.00 (−7.18, −0.98)	0.004 *	−2.14 (−3.85, −0.40)	0.018 *
	Prop. Mediated	0.04 (−0.26, 0.57)	0.598	0.02 (−0.19, 0.35)	0.664	−0.03 (−0.53, 0.18)	0.590
N_CAD	ACME	0.13 (−0.40, 0.86)	0.686	0.35 (−0.42, 1.57)	0.408	0.10 (−0.25, 0.68)	0.628
	ADE	−2.68 (−5.40, −0.17)	0.032 *	−4.54 (−7.54, −1.56)	0.000 *	−2.32 (−4.09, −0.65)	0.006 *
	Total Effect	−2.56 (−5.29, 0.00)	0.050	−4.18 (−7.26, −1.14)	0.002 *	−2.21 (−4.14, −0.47)	0.012 *
	Prop. Mediated	−0.02 (−0.74, 0.28)	0.712	−0.06 (−0.76, 0.10)	0.410	−0.02 (−0.59, 0.13)	0.632
DAP	ACME	−0.03 (−0.75, 0.67)	0.904	−0.15 (−1.98, 1.53)	0.874	−0.03 (−0.64, 0.37)	0.964
	ADE	−2.70 (−5.08, −0.15)	0.036 *	−4.53 (−8.52, −0.09)	0.048 *	−2.36 (−4.37, −0.55)	0.010 *
	Total Effect	−2.73 (−5.20, −0.10)	0.042 *	−4.68 (−9.28, −0.18)	0.040 *	−2.39 (−4.44, −0.58)	0.012 *
	Prop. Mediated	0.01 (−0.45, 0.45)	0.886	0.02 (−0.73, 0.63)	0.842	0.00 (−0.26, 0.29)	0.956
PUT	ACME	0.01 (−0.60, 0.69)	0.942	0.15 (−0.61, 1.16)	0.746	0.36 (−0.18, 1.18)	0.220
	ADE	−2.75 (−5.29, −0.23)	0.032 *	−5.13 (−8.54, −1.78)	0.002 *	−2.55 (−4.19, −0.88)	0.000 *
	Total Effect	−2.74 (−5.30, −0.22)	0.032 *	−4.98 (−8.35, −1.61)	0.002 *	−2.19 (−3.94, −0.37)	0.022 *
	Prop. Mediated	0.00 (−0.41, 0.34)	0.950	−0.01 (−0.36, 0.15)	0.748	−0.14 (−1.35, 0.09)	0.242
CAD	ACME	0.46 (−0.85, 2.33)	0.512	0.40 (−0.74, 2.05)	0.512	0.02 (−0.67, 0.78)	0.944
	ADE	−3.34 (−6.69, −0.20)	0.042 *	−3.13 (−6.29, −0.18)	0.044 *	−2.34 (−4.90, −0.10)	0.042 *
	Total Effect	−2.88 (−6.39, 0.69)	0.098	−2.73 (−6.20, 0.60)	0.106	−2.32 (−4.91, 0.11)	0.054
	Prop. Mediated	−0.09 (−4.08, 1.36)	0.598	−0.08 (−2.70, 1.29)	0.602	0.00 (−0.76, 0.53)	0.978
N_SPD	ACME	0.11 (−0.55, 0.91)	0.730	0.23 (−0.42, 1.28)	0.560	0.35 (−0.17, 1.13)	0.230
	ADE	−2.92 (−5.40, −0.36)	0.028 *	−4.35 (−7.24, −1.31)	0.010 *	−2.51 (−4.31, −0.74)	0.000 *
	Total Effect	−2.81 (−5.33, −0.22)	0.034 *	−4.12 (−7.10, −1.04)	0.014 *	−2.17 (−3.99, −0.31)	0.022 *
	Prop. Mediated	−0.02 (−0.78, 0.26)	0.740	−0.04 (−0.58, 0.15)	0.574	−0.13 (−1.18, 0.08)	0.252
SPD	ACME	−0.08 (−1.06, 0.81)	0.866	−0.05 (−1.14, 0.98)	0.878	−0.03 (−0.48, 0.38)	0.902
	ADE	−2.57 (−4.84, −0.48)	0.020 *	−4.65 (−7.56, −1.63)	0.004 *	−2.09 (−3.66, −0.46)	0.008 *
	Total Effect	−2.65 (−5.06, −0.35)	0.024 *	−4.71 (−7.77, −1.50)	0.006 *	−2.12 (−3.75, −0.48)	0.010 *
	Prop. Mediated	0.02 (−0.70, 0.42)	0.850	0.01 (−0.31, 0.23)	0.876	0.00 (−0.32, 0.26)	0.900
N_SPM	ACME	−1.66 (−3.57, −0.25)	0.020 *	−2.03 (−4.66, −0.04)	0.046 *	−1.04 (−2.31, −0.08)	0.030 *
	ADE	−1.02 (−3.45, 1.67)	0.428	−2.85 (−6.57, 0.84)	0.108	−1.00 (−2.80, 0.93)	0.278
	Total Effect	−2.68 (−4.94, −0.20)	0.040 *	−4.88 (−8.33, −1.53)	0.004 *	−2.05 (−3.73, −0.28)	0.028 *
	Prop. Mediated	0.59 (−0.12, 3.02)	0.060	0.40 (0.00, 1.37)	0.050	0.49 (−0.03, 1.94)	0.058
SPM	ACME	−1.41 (−4.16, 0.23)	0.132	−0.57 (−3.27, 1.53)	0.594	−0.35 (−1.61, 0.64)	0.500
	ADE	−1.34 (−5.21, 2.44)	0.490	−3.45 (−8.12, 1.24)	0.164	−1.50 (−3.74, 0.88)	0.222
	Total Effect	−2.76 (−6.66, 1.18)	0.170	−4.02 (−8.56, 0.19)	0.066	−1.85 (−4.03, 0.34)	0.102
	Prop. Mediated	0.40 (−2.34, 3.29)	0.246	0.10 (−1.02, 1.79)	0.628	0.14 (−1.40, 2.02)	0.558

Data represent estimates from mediation analysis using nonparametric bootstrap (1000 iterations). ACME, ADE, and total effect estimates are presented with 95% confidence intervals (CI). * Nominal *p* < 0.05. *p* values are nominal and were not adjusted for multiplicity. ACME and ADE terminology follows the output of the R mediation package and should be interpreted as exploratory indirect-effect estimates rather than evidence of causal mediation. Analyses were adjusted for age and baseline BMI. Abbreviations: BMI, body mass index; WC, waist circumference; N_PUT, *N*-acetylputrescine; N_CAD, *N*-acetylcadaverine; DAP, 1,3-diaminopropane; PUT, putrescine; CAD, cadaverine; N_SPD, *N*-acetylspermidine; SPD, spermidine; N_SPM, *N*-acetylspermine; SPM, spermine; ACME, average causal mediation effect; ADE, average direct effect; CI, confidence interval; NB, naltrexone/bupropion. Full exploratory indirect-effect estimates are presented in Table S5.

## Data Availability

The datasets generated and analyzed during the current study are available from the corresponding author upon reasonable request. Public data sharing is restricted because the data are derived from an ongoing institutional research project and are maintained within a secure hospital database accessible only to authorized research personnel.

## Supplementary Materials

The following supporting information can be downloaded at: https://www.mdpi.com/article/10.3390/nu18101621/s1, Table S1: Full Dataset of Pre- and Post-intervention Values and Changes in Body Composition, Metabolic Parameters, and Vital Signs Following the 8-Week Intervention; Table S2: Full Dataset of Pre- and Post-intervention Values and Changes in Serum Polyamines Following the 8-Week Intervention; Table S3: Full Exploratory Indirect-Effect Estimates for Serum Polyamines and Serum Metabolic Parameters in All Participants; Table S4: Full Exploratory Indirect-Effect Estimates for Serum Polyamines and Body Composition Parameters in All Participants; Table S5: Full Exploratory Indirect-Effect Estimates for Serum Polyamines and Body Composition in Naltrexone/Bupropion-Treated Participants; Table S6: Full Exploratory Indirect-Effect Estimates for Serum Polyamines and Serum Metabolic Parameters in Naltrexone/Bupropion-Treated Participants.

## Abbreviations

The following abbreviations are used in this manuscript:

ACME	Average Causal Mediation Effect
ADE	Average Direct Effect
BMI	Body Mass Index
CAD	Cadaverine
CI	Confidence Interval
CYP7A1	Cholesterol 7 Alpha-Hydroxylase
DAP	1,3-diaminopropane
DBP	Diastolic Blood Pressure
HDL	High-Density Lipoprotein
HOMA-IR	Homeostatic Model Assessment of Insulin Resistance
HR	Heart Rate
LC–MS	Liquid Chromatography–Mass Spectrometry
LDL	Low-Density Lipoprotein
MeDiet	Mediterranean Diet
NB	Naltrexone/Bupropion
N_CAD	*N*-acetylcadaverine
N_PUT	*N*-acetylputrescine
N_SPD	*N*-acetylspermidine
N_SPM	*N*-acetylspermine
OGF	Opioid Growth Factor
OGFr	Opioid Growth Factor Receptor
PGC-1α	Peroxisome Proliferator-Activated Receptor Gamma Coactivator-1 Alpha
PLS-DA	Partial Least Squares Discriminant Analysis
POMC	Pro-Opiomelanocortin
PUT	Putrescine
QUICKI	Quantitative Insulin Sensitivity Check Index
SBP	Systolic Blood Pressure
SPD	Spermidine
SPM	Spermine
SSAT	Spermidine/Spermine N1-Acetyltransferase
TE	Total Effect
VIP	Variable Importance in Projection
WBC	White Blood Cell
WC	Waist Circumference
WHR	Waist-to-Hip Ratio
