# Supplementary material for: Circulating Polyamines and Metabolic Changes Following a Mediterranean Diet with or Without Naltrexone/Bupropion in Breast Cancer Survivors: An Exploratory Secondary Analysis
Source: Nutrients. 2026 May 20;18(10):1621. doi: 10.3390/nu18101621 (PMC13209824; doi:10.3390/nu18101621)
Supplement: Supplementary file 1 [file nutrients-18-01621-s001.zip › nutrients-4263928-supplementary.pdf]

Table S1. Full Dataset of Pre- and Post-intervention Values and Changes in Body Composition, Metabolic Parameters, and Vital Signs Following the 8-Week Intervention.

Characteristic	Breast Cancer Survivors								Non-Cancer Participants				Pre	Post	Change
	A group (MeDiet Only) (n = 16)				B group (MeDiet+NB) (n = 9)				C group (MeDiet+NB) (n = 16)						
	Pre	Post	change	p-value	Pre	Post	change	p-value	Pre	Post	change	p-value			
Age	55.0 (52.8, 57.5)				56.0 (56.0, 57.0)				55.0 (53.0, 57.5)				0.531		
Height (cm)	158.7 (154.8, 161.2)				157.0 (155.6, 161.5)				156.2 (153.5, 159.2)				0.366		
Body Composition															
Weight (kg)	66.3 (61.8, 68.8)	62.8 (58.5, 67.7)	-1.9 (-3.2, -1.2)	<0.001*	66.5 (63.2, 68.0)	63.2 (60.4, 65.4)	-2.8 (-3.9, -2.3)	0.009*	66.5 (63.0, 73.2)	64.4 (60.5, 71.7)	-2.3 (-3.0, -1.5)	<0.001*	0.658	0.484	0.246
BMI (kg/m^2)	25.6 (24.9, 26.7)	24.7 (23.6, 26.6)	-0.8 (-1.3, -0.5)	<0.001*	27.0 (24.6, 27.6)	25.6 (23.5, 26.5)	-1.1 (-1.6, -0.9)	0.004*	28.3 (25.4, 30.0)	27.4 (24.2, 28.9)	-1.0 (-1.2, -0.6)	<0.001*	0.273	0.223	0.250
WC (cm)	88.5 (85.8, 93.0)	83.0 (82.4, 86.3)	-5.8 (-7.3, -2.4)	0.005*	93.4 (88.0, 94.0)	86.0 (80.0, 89.5)	-5.0 (-7.7, -4.4)	0.013*	92.5 (90.1, 97.4)	90.0 (83.5, 94.8)	-3.3 (-5.9, -0.8)	0.017*	0.187	0.196	0.820
Skeletal muscle (kg)	21.9 (21.3, 22.6)	22.3 (21.1, 22.9)	0.0 (-0.7, 0.6)	0.851	22.3 (21.0, 22.9)	22.2 (21.3, 22.8)	0.1 (-0.3, 0.3)	1.000	21.8 (20.8, 23.2)	22.0 (20.7, 23.0)	0.0 (-0.2, 0.1)	0.726	0.906	0.979	0.999
Fat mass (kg)	25.3 (20.6, 29.5)	22.7 (19.8, 26.2)	-1.9 (-2.4, -1.0)	<0.001*	24.2 (22.9, 28.6)	22.7 (20.4, 24.9)	-2.8 (-3.7, -1.8)	0.004*	27.8 (21.8, 31.7)	25.3 (20.8, 28.7)	-2.3 (-2.9, -0.7)	0.010*	0.618	0.515	0.240
Fat percentage	38.3	37.0	-1.8	0.006*	38.5	35.5	-2.5	0.004*	40.0	37.5	-1.9	0.006*	0.677	0.725	0.584

	(35.2, 42.7)	(32.7, 40.2)	(-2.9, -0.6)		(36.2, 42.4)	(33.7, 38.3)	(-4.0, -1.6)		(34.9, 43.7)	(34.0, 41.2)	(-3.0, -1.1)					
WHR	0.9 (0.9, 1.0)	0.9 (0.9, 1.0)	0.0 (0.0, 0.0)	0.014*	1.0 (0.9, 1.0)	0.9 (0.9, 0.9)	0.0 (-0.1, 0.0)	0.014*	1.0 (0.9, 1.0)	0.9 (0.9, 1.0)	0.0 (0.0, 0.0)	0.009*	0.549	0.792	0.333	
Metabolic Parameters																
WBC (no./μL)	5.2 (4.7, 5.6)	4.5 (4.1, 5.0)	-0.8 (-1.0, -0.2)	0.013*	5.6 (5.0, 6.1)	4.9 (4.3, 6.3)	-0.1 (-0.7, 0.7)	0.820	5.3 (4.8, 6.0)	5.5 (5.0, 6.5)	0.2 (-0.4, 1.1)	0.389	0.535	0.053	0.482	
Fasting glucose (mg/dL)	84.5 (79.0, 92.3)	78.5 (72.0, 90.0)	-11.0 (-13.3, -3.5)	0.004*	85.0 (82.0, 90.0)	75.0 (72.0, 84.0)	-10.0 (-17.0, -4.0)	0.059	88.0 (82.0, 97.0)	78.5 (75.8, 82.3)	-12.0 (-14.3, -6.8)	<0.001*	0.652	0.698	0.736	
Insulin (μIU/mL)	7.0 (5.2, 8.7)	5.5 (3.9, 7.8)	-0.9 (-1.6, -0.1)	0.073	7.5 (5.3, 11.6)	5.5 (4.2, 5.9)	-2.6 (-4.1, -0.7)	0.039*	6.3 (5.1, 9.8)	6.7 (4.8, 7.2)	-1.1 (-2.1, 0.2)	0.088	0.779	0.860	0.143	
HOMA-IR	1.4 (1.1, 2.0)	1.0 (0.7, 1.7)	-0.4 (-0.5, -0.1)	0.034*	1.5 (1.0, 2.7)	0.9 (0.8, 1.5)	-0.7 (-1.1, -0.3)	0.027*	1.5 (1.1, 2.3)	1.3 (0.9, 1.5)	-0.3 (-0.7, -0.1)	0.013*	0.791	0.809	0.170	
QUICKI	0.4 (0.3, 0.4)	0.4 (0.4, 0.4)	0.0 (0.0, 0.0)	0.008*	0.4 (0.3, 0.4)	0.4 (0.4, 0.4)	0.0 (0.0, 0.0)	0.055	0.4 (0.3, 0.4)	0.4 (0.4, 0.4)	0.0 (0.0, 0.0)	0.025*	0.791	0.809	0.473	
Total Cholesterol (mg/dL)	202.0 (182.8, 222.8)	188.5 (179.5, 210.5)	-16.0 (-24.0, 4.5)	0.093	179.0 (152.0, 197.0)	156.5 (133.8, 171.5)	-27.0 (-37.8, -19.3)	0.022*	191.5 (157.8, 212.8)	180.5 (163.3, 191.0)	-17.0 (-21.5, -1.5)	0.020*	0.226	0.018*	0.166	
Triglyceride (mg/dL)	127.0 (102.8, 178.5)	113.5 (78.8, 133.8)	-26.0 (-51.0, 3.0)	0.021*	123.0 (121.0, 150.0)	96.0 (86.8, 135.3)	-38.0 (-44.5, -9.0)	0.161	100.0 (81.8, 143.3)	91.5 (80.8, 168.8)	-2.5 (-18.8, 23.5)	0.959	0.172	0.975	0.026*	
HDL cholesterol (mg/dL)	54.0 (48.5, 65.1)	58.2 (48.4, 62.4)	-1.7 (-2.6, 3.3)	1.000	56.6 (50.6, 60.0)	50.0 (44.9, 53.4)	-0.6 (-2.8, -0.1)	0.183	64.5 (54.2, 71.1)	65.6 (51.3, 69.1)	-1.4 (-5.7, 3.6)	0.744	0.125	0.059	0.561	
LDL cholesterol (mg/dL)	113.5 (99.9, 137.3)	110.7 (101.8, 124.3)	-7.7 (-18.1, 11.6)	0.518	109.9 (63.8, 120.7)	91.1 (66.1, 98.4)	-20.6 (-27.0, -11.9)	0.039*	116.9 (79.4, 128.6)	86.7 (55.2, 108.1)	-10.1 (-29.4, -0.3)	0.008*	0.499	0.023*	0.114	
Vital Signs																

SBP	129.0 (124.3, 138.3)	119.5 (112.0, 130.0)	-8.5 (-19.8, -3.5)	0.012*	121.0 (117.0, 122.0)	115.0 (113.0, 122.0)	-4.0 (-9.0, 6.0)	0.678	125.5 (122.8, 132.8)	122.0 (113.5, 131.3)	-3.5 (-12.3, 0.5)	0.103	0.035*	0.507	0.219
DBP	87.0 (77.8, 95.8)	82.5 (78.0, 89.3)	-4.0 (-12.5, 3.0)	0.155	78.0 (77.0, 81.0)	76.0 (74.0, 80.0)	-2.0 (-5.0, 1.0)	0.400	82.5 (77.0, 87.3)	81.0 (73.0, 86.5)	-3.0 (-4.3, 0.3)	0.040*	0.171	0.368	0.713
HR	78.0 (73.5, 81.8)	73.0 (66.8, 82.0)	-6.5 (-9.3, -1.3)	0.019*	68.0 (65.0, 74.0)	70.0 (65.0, 78.0)	1.0 (0.0, 4.0)	0.175	76.0 (73.0, 85.0)	80.0 (72.0, 86.8)	1.5 (-5.0, 8.8)	0.469	0.048*	0.092	0.019*

Data are presented as medians (interquartile ranges). *P < 0.05 vs. baseline values within each group by Wilcoxon's signed-rank test. Abbreviations: Pre, pre-intervention (baseline); Post, post-intervention (8 weeks); Abbreviations: BMI, body mass index; WC, waist circumference; WHR, waist-to-hip ratio; WBC, white blood cell; HOMA-IR, homeostasis model assessment of insulin resistance; QUICKI, quantitative insulin sensitivity check index; HDL, high-density lipoprotein; LDL, low-density lipoprotein; SBP, systolic blood pressure; DBP, diastolic blood pressure; HR, heart rate; MeDiet, Mediterranean diet; NB, naltrexone/bupropion.

Table S2. Full Dataset of Pre- and Post-intervention Values and Changes in Serum Polyamines Following the 8-Week Intervention.

	Breast Cancer Survivors								Non-Cancer Participants				Pre	Post	Change
	A group (MeDiet alone)				B group (MeDiet+NB)				C group (MeDiet+NB)						
	(n = 16)				(n = 9)				(n = 16)						
	Pre	Post	change	p-value	Pre	Post	change	p-value	Pre	Post	change	p-value			
N_PUT	34.0 (28.5, 58.4)	32.2 (26.5, 46.9)	-4.0 (-16.4, 1.8)	0.175	49.9 (36.3, 64.3)	62.1 (52.3, 85.5)	13.0 (-12.0, 35.6)	0.250	69.0 (61.1, 91.3)	71.9 (60.9, 99.9)	-3.1 (-19.5, 16.4)	0.970	0.001*	<0.001*	0.286
N_CAD	9.3 (6.0, 14.8)	8.1 (4.7, 14.8)	-1.0 (-8.0, 1.3)	0.414	13.5 (9.5, 17.5)	15.0 (7.5, 26.2)	1.7 (-3.4, 6.6)	0.820	12.8 (9.5, 25.0)	13.3 (8.7, 27.8)	-1.3 (-4.4, 4.6)	1.000	0.362	0.122	0.484
DAP	3.4 (3.0, 3.8)	3.9 (3.8, 4.5)	0.4 (0.4, 0.4)	1.000	3.7 (3.0, 4.8)	4.8 (4.5, 7.5)	0.5 (-0.1, 1.4)	0.625	5.3 (4.5, 6.4)	5.8 (3.9, 6.5)	-0.4 (-1.3, 1.3)	0.846	0.018*	0.507	0.802
PUT	29.7 (23.9, 50.5)	35.7 (22.8, 50.7)	-1.0 (-14.1, 9.6)	0.782	34.1 (25.7, 43.8)	47.6 (41.2, 71.5)	15.5 (-1.9, 27.7)	0.074	56.8 (49.9, 69.3)	40.7 (37.6, 96.3)	-10.7 (-19.7, 24.9)	0.903	0.012*	0.144	0.064
CAD	7.0 (4.3, 10.8)	8.9 (4.6, 14.8)	5.8 (-7.9, 11.6)	0.638	13.8 (6.6, 28.4)	25.7 (6.0, 32.8)	9.2 (0.5, 21.7)	0.563	19.9 (14.8, 31.8)	31.3 (10.3, 44.8)	-8.9 (-10.6, 14.8)	0.813	0.046*	0.061	0.848
N_SPD	49.0 (29.5, 72.8)	43.4 (32.8, 56.1)	-2.8 (-22.7, 9.6)	0.274	58.4 (54.4, 91.3)	97.3 (70.9, 125.5)	34.9 (-17.6, 56.2)	0.129	86.3 (78.5, 95.1)	87.4 (65.1, 158.1)	-8.4 (-16.5, 57.9)	0.946	0.005*	<0.001*	0.090
SPD	36.1 (24.4, 49.8)	37.7 (19.5, 69.3)	3.1 (-8.2, 26.6)	0.231	119.1 (69.7, 138.5)	102.3 (91.2, 124.8)	-2.0 (-33.2, 11.1)	0.820	77.2 (53.2, 129.7)	94.3 (64.3, 123.3)	14.7 (-9.5, 32.4)	0.404	0.003*	0.033*	0.617

N_SPM	12.2 (6.2, 16.6)	26.4 (12.9, 45.4)	12.5 (4.7, 35.0)	0.002*	11.6 (9.0, 15.8)	44.4 (12.5, 53.9)	28.6 (5.2, 37.8)	0.004 *	11.6 (8.0, 18.5)	32.0 (14.9, 47.5)	18.5 (4.9, 30.9)	0.002 *	0.971	0.637	0.464
SPM	85.9 (59.3, 113.8)	154.8 (119.5, 303.6)	52.7 (2.5, 196.1)	0.015*	181.1 (130.2, 365.2)	377.3 (287.5, 472.9)	150.5 (24.9, 230.1)	0.625	281.5 (202.7, 322.0)	408.2 (264.4, 467.5)	55.5 (11.7, 210.5)	0.074	0.003*	0.021*	0.946

Data are presented as medians (interquartile ranges). *P < 0.05 vs. baseline values within each group by Wilcoxon's signed-rank test. p values for targeted polyamine analyses are nominal because no formal multiplicity correction was applied. Between-group comparisons were conducted using Quade's rank analysis of covariance, adjusted for age and baseline BMI. Abbreviations: Pre, pre-intervention (baseline); Post, post-intervention (8 weeks); MeDiet, Mediterranean diet; NB, naltrexone/bupropion; BMI, body mass index; N_PUT, N-acetylputrescine; N_CAD, N-acetylcadaverine; DAP, 1,3-diaminopropane; PUT, putrescine; CAD, cadaverine; N_SPD, N-acetylspermidine; SPD, spermidine; N_SPM, N-acetylspermine; SPM, spermine.

Table S3. Full Exploratory Indirect-Effect Estimates for Serum Polyamines and Serum Metabolic Parameters in All Participants.

		WBC (no./μL)		Fasting glucose (mg/dL)		Insulin (μIU/mL)		HOMA-IR		QUICKI		Total Cholesterol (mg/dL)		Triglyceride (mg/dL)		HDL cholesterol (mg/dL)		LDL cholesterol (mg/dL)	
		Estimate (95% CI)	p-value	Estimate (95% CI)	p-value	Estimate (95% CI)	p-value	Estimate (95% CI)	p-value	Estimate (95% CI)	p-value	Estimate (95% CI)	p-value	Estimate (95% CI)	p-value	Estimate (95% CI)	p-value	Estimate (95% CI)	p-value
N_PUT	ACME	0.02 (-0.09, 0.15)	0.712	0.07 (-0.55, 0.84)	0.832	0.04 (-0.16, 0.31)	0.726	0.01 (-0.03, 0.06)	0.776	0.00 (0.00, 0.00)	0.692	-0.67 (-4.55, 2.21)	0.700	1.60 (-7.02, 10.55)	0.716	0.01 (-0.94, 0.99)	0.970	-0.93 (-6.27, 3.74)	0.652
	ADE	-0.22 (-0.75, 0.33)	0.432	-9.92 (-14.34, -5.65)	<0.001*	-1.52 (-2.79, -0.26)	0.010	-0.47 (-0.79, -0.17)	0.002	0.02 (0.01, 0.03)	<0.001	-15.48 (-29.49, -1.78)	0.026*	-22.90 (-44.32, -2.59)	0.032*	0.73 (-5.79, 7.37)	0.854	-14.18 (-28.17, -0.61)	0.042*
	Total Effect	-0.20 (-0.72, 0.38)	0.478	-9.84 (-14.28, -5.53)	<0.001*	-1.48 (-2.79, -0.21)	0.020	-0.46 (-0.80, -0.16)	0.002	0.02 (0.01, 0.03)	<0.001	-16.16 (-31.12, -2.38)	0.022*	-21.29 (-43.95, 1.71)	0.066	0.74 (-5.87, 7.48)	0.848	-15.11 (-29.83, -0.23)	0.048*
	Prop. Mediated	-0.01 (-2.16, 1.94)	0.918	0.00 (-0.09, 0.05)	0.832	-0.01 (-0.39, 0.15)	0.738	-0.01 (-0.23, 0.08)	0.778	-0.02 (-0.28, 0.09)	0.692	0.03 (-0.25, 0.33)	0.698	-0.04 (-1.37, 0.88)	0.770	0.00 (-1.03, 1.44)	0.914	0.05 (-0.49, 0.64)	0.644
N_CAD	ACME	-0.02 (-0.15, 0.07)	0.690	-0.01 (-0.78, 0.71)	0.976	-0.02 (-0.27, 0.19)	0.860	-0.01 (-0.07, 0.04)	0.846	0.00 (0.00, 0.00)	0.938	-0.17 (-2.53, 2.26)	0.854	0.23 (-2.90, 3.81)	0.872	-0.05 (-1.29, 1.04)	0.950	-0.01 (-2.40, 2.44)	0.998
	ADE	-0.18 (-0.71, 0.36)	0.516	-9.15 (-13.95, -4.74)	<0.001*	-1.27 (-2.59, 0.03)	0.058	-0.41 (-0.71, -0.08)	0.012	0.02 (0.00, 0.03)	0.008	-13.72 (-27.67, -0.15)	0.048*	-18.10 (-42.15, 5.29)	0.124	1.14 (-6.06, 7.99)	0.746	-14.13 (-30.01, 0.89)	0.070
	Total Effect	-0.20 (-0.75, 0.33)	0.470	-9.15 (-14.02, -4.78)	<0.001*	-1.29 (-2.64, -0.01)	0.050	-0.42 (-0.72, -0.08)	0.014	0.02 (0.01, 0.03)	0.008	-13.89 (-28.36, -0.11)	0.040*	-17.87 (-42.59, 5.26)	0.124	1.08 (-6.14, 8.14)	0.760	-14.14 (-30.11, 0.96)	0.062
	Prop. Mediated	0.03 (-1.55, 1.92)	0.776	0.00 (-0.09, 0.08)	0.976	0.01 (-0.23, 0.34)	0.858	0.00 (-0.13, 0.19)	0.844	0.00 (-0.17, 0.11)	0.942	0.00 (-0.40, 0.36)	0.842	0.00 (-0.68, 0.39)	0.900	0.00 (-1.23, 1.75)	0.970	0.00 (-0.32, 0.34)	0.984
DAP	ACME	0.00 (-0.23, 0.20)	0.970	-0.10 (-1.93, 1.40)	0.910	-0.07 (-0.83, 0.65)	0.842	-0.02 (-0.19, 0.13)	0.820	0.00 (-0.01, 0.01)	0.812	-0.18 (-4.27, 3.78)	0.944	-1.42 (-16.28, 10.99)	0.798	0.13 (-2.46, 3.44)	0.984	-0.05 (-4.49, 4.77)	0.968

	ADE	-0.12 (-1.03, 0.73)	0.774	-7.17 (-12.92, -1.23)	0.008*	-1.45 (-2.95, -0.04)	0.044*	-0.42 (-0.81, -0.06)	0.024*	0.02 (0.00, 0.03)	0.040*	-13.42 (-31.56, 6.84)	0.182	-5.64 (-34.17, 24.21)	0.716	1.50 (-12.04, 16.59)	0.804	-14.33 (-36.64, 6.75)	0.180
	Total Effect	-0.13 (-1.04, 0.75)	0.766	-7.27 (-13.03, -1.16)	0.016*	-1.52 (-3.23, 0.10)	0.064	-0.44 (-0.85, -0.07)	0.024*	0.02 (0.00, 0.04)	0.072	-13.59 (-31.90, 6.55)	0.190	-7.06 (-39.14, 22.38)	0.668	1.63 (-12.24, 16.72)	0.810	-14.38 (-36.66, 5.95)	0.196
	Prop. Mediated	0.01 (-1.97, 2.69)	0.888	0.00 (-0.33, 0.30)	0.902	0.04 (-1.15, 0.81)	0.810	0.03 (-0.59, 0.55)	0.804	0.05 (-0.96, 1.01)	0.764	0.01 (-0.55, 0.71)	0.914	0.10 (-5.50, 3.88)	0.770	0.01 (-1.67, 1.57)	0.866	0.00 (-0.98, 0.94)	0.908
PUT	ACME	0.06 (-0.05, 0.25)	0.304	-0.26 (-1.32, 0.50)	0.528	0.05 (-0.16, 0.33)	0.652	0.00 (-0.05, 0.05)	0.942	0.00 (0.00, 0.00)	0.626	-1.75 (-6.58, 2.24)	0.350	1.43 (-2.22, 7.58)	0.542	0.20 (-0.82, 1.58)	0.720	-2.22 (-8.51, 2.84)	0.368
	ADE	-0.22 (-0.78, 0.30)	0.426	-9.11 (-13.31, -4.61)	<0.001*	-1.51 (-2.67, -0.33)	0.012*	-0.47 (-0.75, -0.19)	<0.001*	0.02 (0.01, 0.03)	<0.001*	-13.76 (-26.87, -0.46)	0.038*	-20.67 (-42.84, 2.76)	0.070	0.14 (-5.97, 6.16)	0.938	-13.00 (-25.99, 1.30)	0.074
	Total Effect	-0.15 (-0.73, 0.35)	0.580	-9.37 (-13.77, -4.76)	<0.001*	-1.46 (-2.66, -0.26)	0.016*	-0.46 (-0.75, -0.19)	0.004*	0.02 (0.01, 0.03)	<0.001*	-15.51 (-28.49, -1.80)	0.026*	-19.24 (-41.98, 3.62)	0.088	0.34 (-5.67, 6.47)	0.880	-15.22 (-29.12, 0.47)	0.058
	Prop. Mediated	-0.07 (-5.12, 3.15)	0.748	0.02 (-0.06, 0.16)	0.528	-0.02 (-0.41, 0.13)	0.652	0.00 (-0.15, 0.11)	0.946	-0.01 (-0.19, 0.08)	0.626	0.10 (-0.30, 0.60)	0.348	-0.04 (-0.95, 0.30)	0.586	0.01 (-1.50, 1.66)	0.840	0.13 (-0.36, 0.74)	0.362
CAD	ACME	0.03 (-0.11, 0.26)	0.730	-0.22 (-1.74, 0.68)	0.716	-0.02 (-0.35, 0.28)	0.932	-0.01 (-0.09, 0.06)	0.852	0.00 (0.00, 0.00)	0.972	-2.15 (-10.26, 5.99)	0.536	0.73 (-4.43, 7.66)	0.834	-0.25 (-2.13, 1.03)	0.778	-2.10 (-11.00, 4.98)	0.564
	ADE	-0.11 (-0.82, 0.57)	0.762	-9.09 (-13.99, -4.48)	<0.001*	-1.58 (-3.24, 0.06)	0.056	-0.49 (-0.86, -0.11)	0.010*	0.02 (0.01, 0.03)	0.006*	-10.58 (-28.51, 6.49)	0.228	-21.23 (-47.30, 5.25)	0.112	-2.33 (-9.03, 4.42)	0.480	-4.77 (-19.46, 10.22)	0.546
	Total Effect	-0.08 (-0.80, 0.60)	0.832	-9.31 (-14.14, -4.38)	<0.001*	-1.59 (-3.28, 0.06)	0.056	-0.50 (-0.87, -0.11)	0.006*	0.02 (0.01, 0.03)	0.008*	-12.73 (-31.23, 5.41)	0.190	-20.49 (-46.13, 5.91)	0.124	-2.58 (-9.27, 4.44)	0.420	-6.87 (-24.87, 9.00)	0.412
	Prop. Mediated	0.01 (-2.82, 2.44)	0.942	0.01 (-0.10, 0.18)	0.716	0.00 (-0.38, 0.29)	0.948	0.01 (-0.20, 0.22)	0.850	0.00 (-0.21, 0.15)	0.980	0.15 (-2.01, 2.62)	0.546	-0.01 (-0.83, 0.95)	0.886	0.03 (-1.63, 1.91)	0.770	0.21 (-2.26, 3.68)	0.552
N_SPD	ACME	0.05 (-0.06, 0.23)	0.344	-0.37 (-1.56, 0.40)	0.426	0.10 (-0.10, 0.44)	0.394	0.01 (-0.03, 0.08)	0.734	0.00 (0.00, 0.00)	0.478	-1.85 (-6.87, 2.03)	0.348	1.61 (-2.18, 7.40)	0.458	-0.11 (-1.48, 1.00)	0.844	-1.83 (-7.19, 1.95)	0.348
	ADE	-0.23 (-0.77, 0.31)	0.410	-9.02 (-13.20, -4.39)	<0.001*	-1.55 (-2.92, -0.41)	0.010*	-0.47 (-0.78, -0.18)	0.002*	0.02 (0.01, 0.03)	<0.001*	-13.56 (-27.48, 0.16)	0.056	-20.58 (-43.03, 1.30)	0.062	0.55 (-5.86, 6.85)	0.846	-13.05 (-27.16, 0.57)	0.070
	Total Effect	-0.17 (-0.73,	0.518	-9.39	<0.001*	-1.45 (-2.79,	0.022*	-0.46 (-0.77,	0.002*	0.02 (0.01,	<0.001*	-15.41 (-30.64,	0.030*	-18.96 (-41.97,	0.086	0.44 (-6.07,	0.876	-14.88 (-29.46,	0.042*

		0.38)		(-13.46, -4.76)		-0.27)		-0.17)		0.03)		-1.36)		2.62)		6.70)		-0.71)	
	Prop. Mediated	-0.06 (-3.13, 2.68)	0.730	0.03 (-0.05, 0.17)	0.426	-0.05 (-0.67, 0.10)	0.416	-0.01 (-0.22, 0.09)	0.736	-0.03 (-0.28, 0.05)	0.478	0.10 (-0.26, 0.75)	0.358	-0.05 (-1.19, 0.38)	0.500	0.00 (-1.96, 1.44)	0.988	0.10 (-0.27, 0.85)	0.358
SPD	ACME	0.03 (-0.05, 0.19)	0.510	-0.22 (-1.14, 0.52)	0.564	0.03 (-0.15, 0.27)	0.780	0.00 (-0.05, 0.05)	0.980	0.00 (0.00, 0.00)	0.966	-0.63 (-3.50, 1.32)	0.572	-1.53 (-7.86, 2.60)	0.510	0.05 (-1.03, 1.23)	0.932	-0.91 (-4.38, 1.63)	0.484
	ADE	-0.23 (-0.79, 0.32)	0.406	-8.88 (-12.92, -4.66)	<0.001*	-1.36 (-2.49, -0.17)	0.020*	-0.42 (-0.70, -0.13)	<0.001*	0.02 (0.01, 0.03)	0.002*	-13.54 (-26.68, -0.56)	0.040*	-15.02 (-37.27, 4.60)	0.136	0.61 (-5.65, 7.27)	0.866	-12.97 (-26.44, 1.38)	0.070
	Total Effect	-0.20 (-0.79, 0.33)	0.470	-9.10 (-13.14, -4.94)	<0.001*	-1.33 (-2.44, -0.15)	0.026*	-0.42 (-0.70, -0.14)	<0.001*	0.02 (0.01, 0.03)	<0.001*	-14.17 (-27.30, -1.10)	0.030*	-16.55 (-37.73, 3.69)	0.122	0.66 (-5.64, 7.28)	0.854	-13.87 (-27.69, 0.31)	0.054
	Prop. Mediated	-0.03 (-2.44 2.33)	0.760	0.01 (-0.06, 0.14)	0.564	-0.01 (-0.32, 0.15)	0.790	0.00 (-0.15, 0.14)	0.980	0.00 (-0.13, 0.15)	0.966	0.03 (-0.15, 0.40)	0.578	0.06 (-0.91, 0.95)	0.540	0.00 (-1.27, 1.72)	0.954	0.05 (-0.31, 0.50)	0.490
N_SPM	ACME	0.27 (-0.04, 0.63)	0.084	0.50 (-1.90, 2.90)	0.664	0.15 (-0.48, 0.82)	0.666	0.04 (-0.11, 0.21)	0.608	0.00 (-0.01, 0.00)	0.450	-6.31 (-13.98, 1.17)	0.100	2.50 (-8.97, 14.90)	0.656	-2.04 (-5.36, 0.74)	0.152	-3.32 (-12.60, 4.04)	0.402
	ADE	-0.48 (-1.07 0.13)	0.124	-9.91 (-14.52, -5.03)	<0.001*	-1.60 (-2.96, -0.25)	0.018*	-0.50 (-0.83, -0.19)	0.006*	0.02 (0.01, 0.04)	<0.001*	-6.15 (-21.12, 9.68)	0.440	-13.84 (-35.68, 9.43)	0.254	0.94 (-4.71, 7.06)	0.744	-8.86 (-24.95, 7.21)	0.278
	Total Effect	-0.21 (-0.80, 0.34)	0.474	-9.41 (-13.60, -5.00)	<0.001*	-1.45 (-2.60, -0.34)	0.014*	-0.47 (-0.76, -0.18)	0.004*	0.02 (0.01, 0.03)	<0.001*	-12.45 (-26.40, 0.99)	0.082	-11.34 (-30.81, 9.36)	0.296	-1.10 (-6.57, 4.41)	0.658	-12.17 (-25.77, 1.96)	0.098
	Prop. Mediated	-0.54 (-11.50, 9.77)	0.514	-0.05 (-0.37, 0.21)	0.664	-0.09 (-0.93, 0.45)	0.668	-0.08 (-0.63, 0.26)	0.608	-0.13 (-0.77, 0.26)	0.450	0.47 (-1.72, 3.22)	0.178	-0.13 (-3.73, 4.07)	0.772	0.40 (-10.91, 10.61)	0.702	0.22 (-1.33, 2.12)	0.464
SPM	ACME	0.19 (0.00, 0.50)	0.054	0.20 (-1.66, 2.04)	0.808	0.11 (-0.38, 0.62)	0.632	0.02 (-0.10, 0.16)	0.772	0.00 (-0.01, 0.00)	0.308	-4.82 (-11.85, -0.01)	0.046*	-0.86 (-10.67, 8.64)	0.838	-1.08 (-3.38, 0.60)	0.222	-2.56 (-9.09, 1.97)	0.262
	ADE	-0.35 (-1.00, 0.20)	0.248	-9.24 (-14.61, -3.66)	<0.001*	-1.40 (-2.74, -0.14)	0.030*	-0.41 (-0.74, -0.05)	0.028*	0.02 (0.01, 0.04)	0.002*	-9.17 (-22.06, 3.38)	0.164	-23.69 (-55.30, 5.82)	0.120	0.79 (-5.26, 6.87)	0.792	-10.20 (-25.28, 5.28)	0.184
	Total Effect	-0.16 (-0.73, 0.41)	0.602	-9.03 (-14.21, -3.83)	<0.001*	-1.29 (-2.61, -0.07)	0.040*	-0.39 (-0.71, -0.06)	0.024*	0.02 (0.01, 0.04)	<0.001*	-13.99 (-27.15, -0.96)	0.032*	-24.55 (-52.97, 4.03)	0.090	-0.30 (-6.21, 5.83)	0.928	-12.76 (-27.67, 1.94)	0.088
	Prop. Mediated	-0.29 (-7.07, 10.82)	0.616	-0.02 (-0.28, 0.20)	0.808	-0.07 (-1.25, 0.54)	0.660	-0.03 (-0.82, 0.35)	0.772	-0.11 (-0.87, 0.11)	0.308	0.32 (-0.04, 1.52)	0.045*	0.03 (-0.88, 1.38)	0.852	0.04 (-5.02, 7.81)	0.938	0.16 (-0.69, 1.71)	0.326

Data represent estimates from exploratory indirect-effect analyses using the R mediation package with nonparametric bootstrap resampling (1,000 iterations). ACME, ADE, and total effect estimates are presented with 95% confidence intervals (CI). * Nominal $p < 0.05$. p values are nominal and were not adjusted for multiplicity. ACME and ADE terminology follows the output of the R mediation package and should be interpreted as exploratory indirect-effect estimates rather than evidence of causal mediation. Analyses were adjusted for age and baseline BMI. Abbreviations: BMI, body mass index; WBC, white blood cell; HOMA-IR, homeostasis model assessment of insulin resistance; QUICKI, quantitative insulin sensitivity check index; HDL, high-density lipoprotein; LDL, low-density lipoprotein; N_PUT, N-acetylputrescine; N_CAD, N-acetylcadaverine; DAP, 1,3-diaminopropane; PUT, putrescine; CAD, cadaverine; N_SPD, N-acetylspermidine; SPD, spermidine; N_SPM, N-acetylspermine; SPM, spermine; ACME, average causal mediation effect; ADE, average direct effect; CI, confidence interval.

Table S4. Full Exploratory Indirect-Effect Estimates for Serum Polyamines and Body Composition Parameters in All Participants.

		Weight (kg)		BMI (kg/m^2)		WC (cm)		Skeletal muscle (kg)		Fat mass (kg)		Fat percentage		WHR	
		Estimate (95% CI)	p-value	Estimate (95% CI)	p-value	Estimate (95% CI)	p-value	Estimate (95% CI)	p-value	Estimate (95% CI)	p-value	Estimate (95% CI)	p-value	Estimate (95% CI)	p-value
N_PUT	ACME	0.09 (-0.50, 0.77)	0.728	0.09 (-0.30, 0.52)	0.688	0.13 (-0.57, 0.99)	0.690	-0.02 (-0.24, 0.13)	0.788	0.15 (-0.52, 0.93)	0.632	0.15 (-0.58, 0.95)	0.672	0.00 (-0.01, 0.01)	0.656
	ADE	-2.57 (-5.53, 0.59)	0.096	-1.10 (-2.17, -0.02)	0.046 *	-4.28 (-7.58, -1.01)	0.020 *	-0.05 (-1.04, 0.92)	0.948	-2.23 (-4.51, 0.17)	0.070	-2.13 (-4.49, 0.26)	0.076	-0.03 (-0.06, 0.00)	0.070
	Total Effect	-2.47 (-5.59, 0.52)	0.102	-1.01 (-2.16, 0.15)	0.086	-4.15 (-7.37, -0.93)	0.020 *	-0.07 (-1.10, 0.90)	0.886	-2.08 (-4.52, 0.41)	0.100	-1.98 (-4.27, 0.48)	0.114	-0.03 (-0.06, 0.00)	0.094
	Prop. Mediated	-0.01 (-0.84, 0.61)	0.786	-0.06 (-2.93, 0.83)	0.750	-0.02 (-0.43, 0.14)	0.690	0.01 (-1.34, 2.02)	0.938	-0.04 (-1.29, 0.94)	0.688	-0.04 (-1.19, 1.23)	0.750	-0.04 (-1.55, 0.84)	0.722
N_CAD	ACME	-0.04 (-0.64, 0.45)	0.878	-0.02 (-0.26, 0.14)	0.836	0.09 (-0.41, 0.81)	0.776	0.00 (-0.15, 0.15)	0.942	-0.03 (-0.45, 0.34)	0.938	-0.02 (-0.41, 0.32)	0.922	0.00 (-0.01, 0.00)	0.804

	ADE	-2.42 (-5.65, 0.84)	0.146	-0.98 (-2.22, 0.18)	0.110	-4.68 (-7.98, -1.30)	0.004 *	-0.02 (-1.02, 0.97)	0.948	-2.16 (-4.62, 0.30)	0.074	-2.13 (-4.25, 0.12)	0.064	-0.03 (-0.06, 0.01)	0.108
	Total Effect	-2.47 (-5.68, 0.70)	0.138	-1.00 (-2.24, 0.16)	0.104	-4.58 (-7.92, -1.27)	0.010 *	-0.02 (-1.00, 0.97)	0.940	-2.19 (-4.62, 0.27)	0.080	-2.16 (-4.40, 0.12)	0.062	-0.03 (-0.06, 0.01)	0.104
	Prop. Mediated	0.00 (-0.70, 0.38)	0.916	0.01 (-0.47, 0.45)	0.836	-0.01 (-0.27, 0.10)	0.782	0.00 (-1.55, 1.35)	0.942	0.00 (-0.29, 0.39)	0.922	0.00 (-0.25, 0.33)	0.908	0.01 (-0.25, 0.56)	0.804
DAP	ACME	-0.11 (-1.81, 1.41)	0.898	-0.04 (-0.64, 0.43)	0.852	-0.17 (-2.56, 2.10)	0.856	-0.02 (-0.24, 0.21)	0.860	-0.13 (-1.60, 0.88)	0.836	-0.08 (-1.05, 0.70)	0.856	0.00 (-0.01, 0.01)	0.862
	ADE	-2.57 (-6.91, 1.66)	0.250	-1.05 (-3.08, 0.90)	0.310	-4.76 (-10.08, 0.57)	0.082	-0.05 (-0.74, 0.66)	0.894	-2.27 (-5.75, 1.21)	0.224	-1.91 (-4.96, 1.19)	0.228	-0.03 (-0.07, 0.01)	0.170
	Total Effect	-2.68 (-7.24, 1.66)	0.228	-1.09 (-3.23, 0.87)	0.304	-4.93 (-10.73, 0.82)	0.096	-0.07 (-0.75, 0.66)	0.846	-2.40 (-6.05, 1.30)	0.218	-1.99 (-5.03, 1.18)	0.208	-0.03 (-0.07, 0.01)	0.148
	Prop. Mediated	0.03 (-1.77, 1.75)	0.810	0.01 (-1.48, 1.64)	0.824	0.03 (-1.23, 0.91)	0.820	0.01 (-2.12, 3.06)	0.894	0.02 (-1.57, 1.32)	0.818	0.02 (-1.10, 1.46)	0.828	0.01 (-0.95, 1.08)	0.838
PUT	ACME	-0.03 (-0.66, 0.46)	0.942	0.06 (-0.12, 0.33)	0.546	0.04 (-0.50, 0.71)	0.862	-0.09 (-0.38, 0.09)	0.376	0.14 (-0.21, 0.70)	0.534	0.20 (-0.22, 0.90)	0.410	0.00 (0.00, 0.01)	0.504
	ADE	-2.48 (-5.27, 0.56)	0.122	-1.04 (-2.23, 0.09)	0.068	-4.88 (-8.48, -1.35)	0.006 *	0.02 (-0.89, 0.93)	0.956	-2.17 (-4.58, 0.22)	0.068	-2.19 (-4.52, 0.14)	0.066	-0.03 (-0.06, 0.00)	0.068
	Total Effect	-2.50 (-5.42, 0.52)	0.120	-0.98 (-2.18, 0.13)	0.104	-4.84 (-8.32, -1.26)	0.006 *	-0.06 (-0.95, 0.90)	0.910	-2.03 (-4.56, 0.53)	0.088	-1.99 (-4.38, 0.41)	0.100	-0.03 (-0.06, 0.00)	0.098

	Prop. Mediated	0.00 (-0.41, 0.58)	0.926	-0.04 (-0.85, 0.77)	0.614	0.00 (-0.20, 0.13)	0.860	0.03 (-4.36, 2.92)	0.874	-0.03 (-0.98, 0.37)	0.578	-0.06 (-1.41, 0.84)	0.486	-0.04 (-0.87, 0.40)	0.574
CAD	ACME	0.11 (-0.62, 1.09)	0.774	-0.06 (-0.44, 0.21)	0.692	0.29 (-0.81, 1.78)	0.656	0.09 (-0.21, 0.54)	0.604	-0.06 (-0.72, 0.48)	0.866	-0.11 (-0.88, 0.48)	0.776	0.00 (-0.01, 0.01)	0.718
	ADE	-2.60 (-6.76, 1.75)	0.204	-0.92 (-2.41, 0.62)	0.238	-3.88 (-8.34, 0.56)	0.090	-0.28 (-1.59, 1.04)	0.724	-1.83 (-4.51, 0.90)	0.216	-1.52 (-4.34, 1.15)	0.318	-0.02 (-0.06, 0.02)	0.334
	Total Effect	-2.49 (-6.65, 1.79)	0.220	-0.98 (-2.48, 0.58)	0.208	-3.59 (-8.25, 1.08)	0.126	-0.19 (-1.58, 1.15)	0.800	-1.89 (-4.58, 0.88)	0.202	-1.63 (-4.46, 1.13)	0.284	-0.02 (-0.06, 0.02)	0.288
	Prop. Mediated	-0.01 (-1.27, 0.72)	0.830	0.02 (-1.12, 0.99)	0.728	-0.03 (-1.52, 1.23)	0.742	0.00 (-3.32, 3.41)	0.972	0.01 (-0.70, 0.90)	0.864	0.02 (-1.14, 1.43)	0.792	0.03 (-1.10, 1.47)	0.746
N_SPD	ACME	0.12 (-0.41, 0.81)	0.664	0.07 (-0.10, 0.38)	0.510	0.28 (-0.25, 1.22)	0.408	-0.03 (-0.24, 0.14)	0.718	0.20 (-0.21, 0.91)	0.458	0.21 (-0.24, 0.92)	0.422	0.00 (0.00, 0.01)	0.502
	ADE	-2.66 (-5.65, 0.67)	0.108	-1.10 (-2.26, 0.13)	0.076	-4.49 (-7.91, -0.82)	0.018*	-0.07 (-1.00, 0.95)	0.888	-2.24 (-4.68, 0.34)	0.102	-2.22 (-4.35, 0.00)	0.050	-0.03 (-0.06, 0.00)	0.078
	Total Effect	-2.54 (-5.53, 0.65)	0.106	-1.03 (-2.18, 0.16)	0.096	-4.21 (-7.73, -0.68)	0.018*	-0.10 (-1.04, 0.94)	0.848	-2.04 (-4.49, 0.53)	0.124	-2.01 (-4.22, 0.29)	0.080	-0.03 (-0.06, 0.01)	0.128
	Prop. Mediated	-0.03 (-0.85, 0.34)	0.678	-0.04 (-0.77, 0.37)	0.546	-0.05 (-0.55, 0.09)	0.418	0.01 (-2.17, 1.73)	0.930	-0.05 (-1.08, 0.67)	0.538	-0.06 (-1.20, 0.33)	0.478	-0.04 (-1.20, 0.99)	0.590
SPD	ACME	-0.23 (-1.04, 0.31)	0.424	-0.01 (-0.22, 0.18)	0.940	-0.17 (-1.04, 0.37)	0.542	-0.05 (-0.27, 0.09)	0.532	-0.13 (-0.70, 0.24)	0.528	-0.09 (-0.52, 0.21)	0.656	0.00 (-0.01, 0.00)	0.866
	ADE	-2.28	0.108	-0.99	0.090	-4.51	0.002*	-0.02	0.982	-1.90	0.114	-1.97	0.082	-0.03	0.104

		(-5.32, 0.35)		(-2.14, 0.12)		(-7.51, -1.30)		(-0.88, 0.80)		(-4.31, 0.43)		(-4.16, 0.21)		(-0.05, 0.01)	
	Total Effect	-2.51 (-5.49, 0.28)	0.082	-0.99 (-2.15, 0.14)	0.088	-4.68 (-7.74, -1.54)	0.006*	-0.07 (-0.93, 0.78)	0.922	-2.04 (-4.41, 0.33)	0.100	-2.06 (-4.23, 0.17)	0.070	-0.03 (-0.05, 0.00)	0.096
	Prop. Mediated	0.07 (-0.37, 0.84)	0.458	0.00 (-0.57, 0.36)	0.940	0.02 (-0.09, 0.30)	0.540	0.01 (-4.97, 2.38)	0.946	0.04 (-0.38, 0.70)	0.540	0.02 (-0.26, 0.50)	0.666	0.01 (-0.47, 0.50)	0.850
N_SPM	ACME	0.33 (-1.25, 2.09)	0.694	0.45 (-0.21, 1.25)	0.184	-0.49 (-2.63, 1.65)	0.614	0.07 (-0.47, 0.62)	0.794	0.22 (-1.13, 1.50)	0.736	0.20 (-1.04, 1.44)	0.748	0.01 (-0.01, 0.03)	0.204
	ADE	-2.76 (-6.15, 0.76)	0.128	-1.43 (-2.81, -0.09)	0.030*	-4.35 (-8.43, -0.39)	0.026*	-0.15 (-1.20, 0.86)	0.804	-2.23 (-4.92, 0.54)	0.130	-2.13 (-4.71, 0.58)	0.098	-0.04 (-0.07, 0.00)	0.034*
	Total Effect	-2.43 (-5.53, 0.72)	0.130	-0.98 (-2.13, 0.28)	0.104	-4.84 (-8.43, -1.25)	0.012*	-0.08 (-0.95, 0.84)	0.848	-2.01 (-4.44, 0.46)	0.118	-1.93 (-4.04, 0.47)	0.112	-0.03 (-0.06, 0.00)	0.092
	Prop. Mediated	-0.10 (-3.08, 2.54)	0.736	-0.38 (-4.55, 2.02)	0.268	0.10 (-0.51, 0.75)	0.614	0.00 (-5.35, 8.48)	0.998	-0.10 (-2.53, 1.44)	0.762	-0.08 (-2.01, 2.03)	0.764	-0.36 (-4.03, 2.98)	0.288
SPM	ACME	0.26 (-0.97, 1.72)	0.698	0.52 (0.02, 1.31)	0.038*	0.88 (-0.42, 2.72)	0.202	-0.12 (-0.61, 0.38)	0.534	0.44 (-0.54, 1.68)	0.382	0.62 (-0.31, 1.92)	0.204	0.01 (-0.01, 0.02)	0.290
	ADE	-2.84 (-6.57, 0.88)	0.146	-1.53 (-3.15, -0.13)	0.036*	-5.33 (-9.38, -1.32)	0.006*	-0.04 (-1.30, 1.26)	0.958	-2.44 (-5.43, 0.43)	0.116	-2.42 (-5.29, 0.21)	0.074	-0.03 (-0.07, 0.01)	0.132
	Total Effect	-2.58 (-6.20, 1.02)	0.172	-1.01 (-2.57, 0.42)	0.170	-4.45 (-8.46, -0.30)	0.042*	-0.16 (-1.43, 1.07)	0.800	-2.01 (-5.00, 0.86)	0.176	-1.80 (-4.59, 0.81)	0.198	-0.02 (-0.06, 0.02)	0.234
	Prop. Mediated	-0.05 (-1.64, 1.26)	0.774	-0.38 (-7.49, 4.35)	0.208	-0.16 (-1.54, 0.34)	0.244	0.04 (-6.44, 4.39)	0.918	-0.15 (-2.29, 1.81)	0.502	-0.22 (-3.90, 3.94)	0.370	-0.19 (-3.14, 2.87)	0.460

Data represent estimates from exploratory indirect-effect analyses using the R mediation package with nonparametric bootstrap resampling (1,000 iterations). ACME, ADE, and total effect estimates are presented with 95% confidence intervals (CI). * Nominal p < 0.05. p values are nominal and were not adjusted for multiplicity. ACME and ADE terminology follows the output of the R mediation package and should be interpreted as exploratory indirect-effect estimates rather than evidence of causal mediation. Analyses were adjusted for age and baseline BMI. Abbreviations: BMI, body mass index; WC, waist circumference; WHR, waist-to-hip ratio; N_PUT, N-acetylputrescine; N_CAD, N-acetylcadaverine; DAP, 1,3-diaminopropane; PUT, putrescine; CAD, cadaverine; N_SPD, N-acetylspermidine; SPD, spermidine; N_SPM, N-acetylspermine; SPM, spermine; ACME, average causal mediation effect; ADE, average direct effect; CI, confidence interval.

Table S5. Full Exploratory Indirect-Effect Estimates for Serum Polyamines and Body Composition in Naltrexone/Bupropion-Treated Participants.

		Weight (kg)		BMI (kg/m^2)		WC (cm)		Skeletal muscle (kg)		Fat mass (kg)		Fat percentage		WHR	
		Estimate (95% CI)	p-value	Estimate (95% CI)	p-value	Estimate (95% CI)	p-value	Estimate (95% CI)	p-value	Estimate (95% CI)	p-value	Estimate (95% CI)	p-value	Estimate (95% CI)	p-value
N_PUT	ACME	-0.19 (-1.05, 0.39)	0.588	0.33 (-0.33, 1.26)	0.320	-0.17 (-1.11, 0.60)	0.660	-0.19 (-0.78, 0.18)	0.410	0.12 (-0.24, 0.67)	0.584	0.31 (-0.30, 1.21)	0.356	0.00 (-0.01, 0.01)	0.822
	ADE	-2.54 (-5.12, -0.01)	0.048 *	-1.40 (-3.08, 0.27)	0.106	-3.83 (-6.91, -0.94)	0.006 *	0.15 (-1.08, 1.53)	0.860	-2.26 (-3.99, -0.50)	0.014 *	-2.49 (-4.70, -0.32)	0.026 *	-0.03 (-0.06, 0.00)	0.076
	Total Effect	-2.73 (-5.38, -0.15)	0.042 *	-1.06 (-2.83, 0.71)	0.216	-4.00 (-7.18, -0.98)	0.004 *	-0.03 (-1.30, 1.37)	0.972	-2.14 (-3.85, -0.40)	0.018 *	-2.18 (-4.56, 0.09)	0.062	-0.03 (-0.06, 0.00)	0.068

	Prop. Mediated	0.04 (-0.26, 0.57)	0.598	-0.16 (-3.80, 2.45)	0.520	0.02 (-0.19, 0.35)	0.664	0.05 (-3.97, 5.17)	0.866	-0.03 (-0.53, 0.18)	0.590	-0.10 (-1.97, 0.27)	0.410	-0.01 (-0.88, 0.37)	0.822
N_CAD	ACME	0.13 (-0.40, 0.86)	0.686	-0.13 (-0.69, 0.26)	0.522	0.35 (-0.42, 1.57)	0.408	0.02 (-0.25, 0.33)	0.868	0.10 (-0.25, 0.68)	0.628	0.08 (-0.31, 0.70)	0.688	0.00 (0.00, 0.01)	0.918
	ADE	-2.68 (-5.40, -0.17)	0.032*	-0.91 (-2.57, 0.76)	0.328	-4.54 (-7.54, -1.56)	0.000*	-0.03 (-1.31, 1.26)	0.954	-2.32 (-4.09, -0.65)	0.006*	-2.39 (-4.42, -0.59)	0.016*	-0.03 (-0.06, 0.00)	0.024*
	Total Effect	-2.56 (-5.29, 0.00)	0.050	-1.05 (-2.74, 0.68)	0.278	-4.18 (-7.26, -1.14)	0.002*	-0.02 (-1.37, 1.25)	0.974	-2.21 (-4.14, -0.47)	0.012*	-2.31 (-4.28, -0.45)	0.018*	-0.03 (-0.06, 0.00)	0.024*
	Prop. Mediated	-0.02 (-0.74, 0.28)	0.712	0.06 (-1.21, 1.54)	0.580	-0.06 (-0.76, 0.10)	0.410	0.00 (-2.16, 1.96)	0.950	-0.02 (-0.59, 0.13)	0.632	-0.02 (-0.56, 0.18)	0.702	0.00 (-0.43, 0.27)	0.922
DAP	ACME	-0.03 (-0.75, 0.67)	0.904	-0.02 (-0.60, 0.45)	0.960	-0.15 (-1.98, 1.53)	0.874	-0.01 (-0.24, 0.19)	0.924	-0.03 (-0.64, 0.37)	0.964	-0.01 (-0.47, 0.37)	0.996	0.00 (-0.01, 0.01)	0.978
	ADE	-2.70 (-5.08, -0.15)	0.036*	-1.08 (-3.15, 1.07)	0.306	-4.53 (-8.52, -0.09)	0.048*	-0.11 (-0.84, 0.59)	0.758	-2.36 (-4.37, -0.55)	0.010*	-2.08 (-4.05, -0.10)	0.046*	-0.03 (-0.06, 0.00)	0.032*
	Total Effect	-2.73 (-5.20, -0.10)	0.042*	-1.11 (-3.22, 1.03)	0.320	-4.68 (-9.28, -0.18)	0.040*	-0.11 (-0.85, 0.63)	0.732	-2.39 (-4.44, -0.58)	0.012*	-2.09 (-4.05, -0.08)	0.040*	-0.03 (-0.06, 0.00)	0.030*
	Prop. Mediated	0.01 (-0.45, 0.45)	0.886	0.01 (-0.98, 1.18)	0.932	0.02 (-0.73, 0.63)	0.842	0.02 (-2.04, 1.97)	0.856	0.00 (-0.26, 0.29)	0.956	0.00 (-0.31, 0.29)	0.992	0.00 (-0.41, 0.25)	0.992
PUT	ACME	0.01 (-0.60, 0.69)	0.942	0.05 (-0.36, 0.58)	0.792	0.15 (-0.61, 1.16)	0.746	-0.20 (-0.71, 0.12)	0.298	0.36 (-0.18, 1.18)	0.220	0.55 (-0.28, 1.75)	0.220	0.01 (0.00, 0.02)	0.270
	ADE	-2.75	0.032*	-1.14	0.200	-5.13	0.002*	0.11	0.852	-2.55	0.000*	-2.72	0.006*	-0.03	0.016*

		(-5.29, -0.23)		(-2.94, 0.63)		(-8.54, -1.78)		(-1.20, 1.30)		(-4.19, -0.88)		(-4.81, -0.66)		(-0.06, -0.01)	
	Total Effect	-2.74 (-5.30, -0.22)	0.032 *	-1.09 (-2.95, 0.79)	0.234	-4.98 (-8.35, -1.61)	0.002 *	-0.09 (-1.38, 1.15)	0.870	-2.19 (-3.94, -0.37)	0.022 *	-2.16 (-4.34, -0.05)	0.048 *	-0.03 (-0.06, 0.00)	0.054
	Prop. Mediated	0.00 (-0.41, 0.34)	0.950	-0.01 (-1.83, 0.92)	0.882	-0.01 (-0.36, 0.15)	0.748	0.10 (-3.41, 4.67)	0.796	-0.14 (-1.35, 0.09)	0.242	-0.21 (-2.45, 0.24)	0.268	-0.13 (-1.86, 0.31)	0.320
CAD	ACME	0.46 (-0.85, 2.33)	0.512	-0.13 (-0.95, 0.44)	0.682	0.40 (-0.74, 2.05)	0.512	0.25 (-0.53, 1.26)	0.504	0.02 (-0.67, 0.78)	0.944	-0.20 (-1.37, 0.65)	0.722	0.00 (-0.02, 0.01)	0.612
	ADE	-3.34 (-6.69, -0.20)	0.042 *	-1.07 (-3.39, 1.19)	0.404	-3.13 (-6.29, -0.18)	0.044 *	-0.50 (-2.53, 1.42)	0.624	-2.34 (-4.90, -0.10)	0.042 *	-1.79 (-5.02, 1.58)	0.294	-0.02 (-0.07, 0.02)	0.302
	Total Effect	-2.88 (-6.39, 0.69)	0.098	-1.19 (-3.52, 1.11)	0.324	-2.73 (-6.20, 0.60)	0.106	-0.25 (-2.47, 1.85)	0.824	-2.32 (-4.91, 0.11)	0.054	-1.98 (-5.19, 1.35)	0.244	-0.03 (-0.07, 0.02)	0.274
	Prop. Mediated	-0.09 (-4.08, 1.36)	0.598	0.04 (-1.48, 2.48)	0.702	-0.08 (-2.70, 1.29)	0.602	0.04 (-4.71, 4.32)	0.900	0.00 (-0.76, 0.53)	0.978	0.04 (-1.58, 1.77)	0.754	0.07 (-1.03, 1.73)	0.626
N_SPD	ACME	0.11 (-0.55, 0.91)	0.730	0.04 (-0.48, 0.65)	0.888	0.23 (-0.42, 1.28)	0.560	-0.12 (-0.57, 0.19)	0.534	0.35 (-0.17, 1.13)	0.230	0.49 (-0.21, 1.50)	0.220	0.00 (0.00, 0.02)	0.376
	ADE	-2.92 (-5.40, -0.36)	0.028 *	-1.12 (-3.02, 0.71)	0.264	-4.35 (-7.24, -1.31)	0.010 *	0.06 (-1.21, 1.50)	0.942	-2.51 (-4.31, -0.74)	0.000 *	-2.57 (-4.55, -0.48)	0.022 *	-0.03 (-0.06, 0.00)	0.048 *
	Total Effect	-2.81 (-5.33, -0.22)	0.034 *	-1.08 (-3.02, 0.76)	0.292	-4.12 (-7.10, -1.04)	0.014 *	-0.06 (-1.33, 1.31)	0.904	-2.17 (-3.99, -0.31)	0.022 *	-2.08 (-4.18, 0.09)	0.060	-0.03 (-0.06, 0.00)	0.064
	Prop. Mediated	-0.02 (-0.78, 0.26)	0.740	0.00 (-2.05, 2.35)	0.988	-0.04 (-0.58, 0.15)	0.574	0.02 (-3.81, 3.40)	0.938	-0.13 (-1.18, 0.08)	0.252	-0.19 (-3.51, 0.29)	0.268	-0.08 (-1.55, 0.44)	0.424

SPD	ACME	-0.08 (-1.06, 0.81)	0.866	-0.01 (-0.29, 0.27)	1.000	-0.05 (-1.14, 0.98)	0.878	-0.02 (-0.38, 0.31)	0.870	-0.03 (-0.48, 0.38)	0.902	-0.01 (-0.39, 0.29)	0.960	0.00 (0.00, 0.00)	0.972
	ADE	-2.57 (-4.84, -0.48)	0.020*	-1.00 (-2.58, 0.53)	0.212	-4.65 (-7.56, -1.63)	0.004*	-0.02 (-1.10, 1.11)	0.964	-2.09 (-3.66, -0.46)	0.008*	-2.08 (-4.10, -0.21)	0.038*	-0.03 (-0.05, 0.00)	0.030*
	Total Effect	-2.65 (-5.06, -0.35)	0.024*	-1.00 (-2.66, 0.54)	0.222	-4.71 (-7.77, -1.50)	0.006*	-0.04 (-1.20, 1.12)	0.954	-2.12 (-3.75, -0.48)	0.010*	-2.09 (-4.13, -0.23)	0.036*	-0.03 (-0.05, 0.00)	0.026*
	Prop. Mediated	0.02 (-0.70, 0.42)	0.850	0.00 (-0.52, 1.29)	0.942	0.01 (-0.31, 0.23)	0.876	0.03 (-2.83, 2.76)	0.844	0.00 (-0.32, 0.26)	0.900	0.00 (-0.19, 0.28)	0.960	0.00 (-0.25, 0.23)	0.974
N_SPM	ACME	-1.66 (-3.57, -0.25)	0.020*	0.26 (-0.66, 1.38)	0.616	-2.03 (-4.66, -0.04)	0.046*	-0.29 (-1.08, 0.46)	0.426	-1.04 (-2.31, -0.08)	0.030*	-0.60 (-1.87, 0.59)	0.290	0.01 (-0.01, 0.03)	0.252
	ADE	-1.02 (-3.45, 1.67)	0.428	-1.31 (-3.24, 0.60)	0.170	-2.85 (-6.57, 0.84)	0.108	0.21 (-1.19, 1.61)	0.780	-1.00 (-2.80, 0.93)	0.278	-1.39 (-3.64, 1.02)	0.226	-0.04 (-0.07, -0.01)	0.016*
	Total Effect	-2.68 (-4.94, -0.20)	0.040*	-1.05 (-2.77, 0.64)	0.230	-4.88 (-8.33, -1.53)	0.004*	-0.08 (-1.34, 1.19)	0.882	-2.05 (-3.73, -0.28)	0.028*	-1.99 (-4.05, 0.03)	0.056	-0.03 (-0.06, 0.00)	0.028*
	Prop. Mediated	0.59 (-0.12, 3.02)	0.060	-0.15 (-4.11, 4.96)	0.722	0.40 (0.00, 1.37)	0.050	0.02 (-5.60, 8.60)	0.980	0.49 (-0.03, 1.94)	0.058	0.27 (-0.66, 2.66)	0.326	-0.26 (-2.65, 0.48)	0.280
SPM	ACME	-1.41 (-4.16, 0.23)	0.132	0.56 (-0.48, 2.13)	0.362	-0.57 (-3.27, 1.53)	0.594	-0.67 (-2.21, 0.30)	0.198	-0.35 (-1.61, 0.64)	0.500	0.45 (-0.94, 2.15)	0.484	0.01 (-0.01, 0.03)	0.536
	ADE	-1.34 (-5.21, 2.44)	0.490	-1.61 (-4.25, 1.05)	0.272	-3.45 (-8.12, 1.24)	0.164	0.48 (-1.88, 2.80)	0.676	-1.50 (-3.74, 0.88)	0.222	-2.34 (-5.15, 0.57)	0.122	-0.03 (-0.07, 0.01)	0.184
	Total Effect	-2.76	0.170	-1.05	0.428	-4.02	0.066	-0.19	0.858	-1.85	0.102	-1.88	0.184	-0.02	0.232

	(-6.66, 1.18)		(-3.49, 1.40)		(-8.56, 0.19)		(-2.45, 2.14)		(-4.03, 0.34)		(-4.62, 0.85)		(-0.06, 0.02)	
Prop. Mediated	0.40 (-2.34, 3.29)	0.246	-0.19 (-7.61, 5.48)	0.646	0.10 (-1.02, 1.79)	0.628	0.16 (-10.10, 10.73)	0.880	0.14 (-1.40, 2.02)	0.558	-0.13 (-3.71, 2.93)	0.592	-0.11 (-4.72, 2.57)	0.680

Data represent estimates from exploratory indirect-effect analyses using the R mediation package with nonparametric bootstrap resampling (1,000 iterations). ACME, ADE, and total effect estimates are presented with 95% confidence intervals (CI). * Nominal p < 0.05. p values are nominal and were not adjusted for multiplicity. ACME and ADE terminology follows the output of the R mediation package and should be interpreted as exploratory indirect-effect estimates rather than evidence of causal mediation. Analyses were adjusted for age and baseline BMI. Abbreviations: BMI, body mass index; WC, waist circumference; WHR, waist-to-hip ratio; N_PUT, N-acetylputrescine; N_CAD, N-acetylcadaverine; DAP, 1,3-diaminopropane; PUT, putrescine; CAD, cadaverine; N_SPD, N-acetylspermidine; SPD, spermidine; N_SPM, N-acetylspermine; SPM , spermine; ACME, average causal mediation effect; ADE, average direct effect; CI, confidence interval.

Table S6. Full Exploratory Indirect-Effect Estimates for Serum Polyamines and Serum Metabolic Parameters in Naltrexone/Bupropion-Treated Participants.

		WBC (no./μL)		Fasting glucose (mg/dL)		Insulin (μIU/mL)		HOMA-IR		QUICKI		Total Cholesterol (mg/dL)		Triglyceride (mg/dL)		HDL cholesterol (mg/dL)		LDL cholesterol (mg/dL)	
		Estimate (95% CI)	p-value	Estimate (95% CI)	p-value	Estimate (95% CI)	p-value	Estimate (95% CI)	p-value	Estimate (95% CI)	p-value	Estimate (95% CI)	p-value	Estimate (95% CI)	p-value	Estimate (95% CI)	p-value	Estimate (95% CI)	p-value
N_PUT	ACME	0.08 (-0.09, 0.35)	0.436	-0.21 (-1.82, 0.95)	0.760	0.05 (-0.37, 0.58)	0.802	0.01 (-0.09, 0.11)	0.932	0.00 (0.00, 0.00)	0.752	-1.05 (-7.43, 3.33)	0.672	6.39 (-6.23, 22.84)	0.302	-0.40 (-3.47, 1.99)	0.740	-1.26 (-8.00, 3.29)	0.650
	ADE	-0.07 (-0.80, 0.66)	0.874	-10.81 (-15.56, -5.41)	0.000 *	-2.03 (-3.73, -0.31)	0.020 *	-0.63 (-1.02, -0.26)	0.002 *	0.02 (0.01, 0.04)	0.000 *	-19.84 (-39.32, 0.96)	0.066	-13.97 (-37.46, 12.00)	0.240	1.19 (-10.02, 12.77)	0.846	-24.56 (-45.75, -4.44)	0.014 *
	Total Effect	0.01	0.966	-11.02	0.000 *	-1.99	0.020 *	-0.62	0.002 *	0.02	0.000 *	-20.89	0.066	-7.58	0.558	0.79	0.902	-25.83	0.012 *

		(-0.72, 0.74)		(-16.04, -5.59)		(-3.75, -0.27)		(-1.01, -0.25)		(0.01, 0.04)		(-40.16, 0.68)		(-33.76, 22.69)		(-10.34, 12.02)		(-47.19, -5.51)	
	Prop. Mediated	0.02 (-3.40, 3.84)	0.934	0.01 (-0.09, 0.18)	0.760	-0.01 (-0.53, 0.23)	0.810	0.00 (-0.23, 0.17)	0.930	-0.01 (-0.26, 0.13)	0.752	0.03 (-0.27, 0.57)	0.666	-0.11 (-8.26, 7.10)	0.820	0.00 (-2.69, 2.02)	0.998	0.03 (-0.24, 0.38)	0.658
N_CAD	ACME	-0.07 (-0.35, 0.09)	0.444	-0.13 (-1.45, 0.89)	0.804	0.01 (-0.37, 0.40)	0.930	0.00 (-0.08, 0.08)	0.966	0.00 (0.00, 0.00)	0.718	0.88 (-2.93, 6.68)	0.692	2.19 (-3.54, 9.90)	0.448	-0.46 (-3.89, 1.81)	0.720	1.36 (-2.81, 8.19)	0.558
	ADE	0.01 (-0.69, 0.68)	0.920	-9.86 (-15.01, -4.94)	0.000*	-1.60 (-3.19, 0.06)	0.056	-0.51 (-0.85, -0.15)	0.006*	0.02 (0.00, 0.03)	0.004*	-17.43 (-36.68, 1.44)	0.086	-5.88 (-27.07, 17.22)	0.598	1.58 (-9.14, 12.30)	0.800	-23.88 (-43.73, -3.81)	0.024*
	Total Effect	-0.07 (-0.79, 0.63)	0.914	-9.99 (-15.07, -4.92)	0.000*	-1.58 (-3.20, 0.06)	0.054	-0.51 (-0.85, -0.15)	0.006*	0.02 (0.00, 0.03)	0.004*	-16.55 (-35.30, 3.02)	0.114	-3.69 (-26.40, 20.72)	0.748	1.11 (-9.96, 12.00)	0.844	-22.52 (-42.88, -1.68)	0.026*
	Prop. Mediated	0.02 (-3.71, 3.50)	0.906	0.01 (-0.10, 0.16)	0.804	0.00 (-0.42, 0.32)	0.944	0.00 (-0.24, 0.16)	0.960	-0.01 (-0.61, 0.13)	0.718	-0.02 (-0.83, 0.71)	0.754	-0.01 (-4.80, 3.15)	0.956	0.00 (-3.45, 2.49)	1.000	-0.03 (-0.98, 0.17)	0.564
DAP	ACME	-0.01 (-0.29, 0.21)	0.918	-0.08 (-1.80, 1.39)	0.930	-0.05 (-0.79, 0.72)	0.860	-0.01 (-0.18, 0.15)	0.880	0.00 (-0.01, 0.01)	0.832	-0.08 (-4.01, 3.97)	0.962	-0.63 (-13.63, 11.85)	0.886	0.08 (-3.41, 4.07)	0.958	0.05 (-4.82, 5.14)	0.998
	ADE	-0.11 (-1.16, 0.94)	0.852	-8.35 (-14.25, -2.61)	0.002*	-1.48 (-2.95, 0.09)	0.054	-0.44 (-0.79, -0.10)	0.012*	0.02 (0.00, 0.03)	0.032*	-13.93 (-31.76, 3.30)	0.124	-2.89 (-37.95, 32.07)	0.854	1.68 (-14.57, 17.14)	0.846	-15.63 (-36.12, 4.90)	0.150
	Total Effect	-0.12 (-1.20, 0.94)	0.840	-8.43 (-14.50, -2.70)	0.006*	-1.53 (-3.14, 0.21)	0.086	-0.46 (-0.83, -0.06)	0.018*	0.02 (0.00, 0.03)	0.042*	-14.01 (-32.00, 3.47)	0.124	-3.52 (-42.27, 33.25)	0.848	1.76 (-14.75, 17.97)	0.834	-15.58 (-36.52, 6.11)	0.158
	Prop. Mediated	0.01 (-2.50, 2.24)	0.902	0.00 (-0.27, 0.21)	0.924	0.03 (-1.35, 0.82)	0.814	0.02 (-0.87, 0.44)	0.862	0.03 (-0.78, 0.73)	0.798	0.00 (-0.56, 0.62)	0.918	0.05 (-5.02, 3.18)	0.822	0.02 (-1.80, 1.44)	0.820	0.00 (-0.68, 0.71)	0.960
PUT	ACME	0.09 (-0.07, 0.37)	0.348	-0.14 (-1.70, 1.09)	0.800	0.20 (-0.16, 0.80)	0.364	0.04 (-0.04, 0.16)	0.434	0.00 (-0.01, 0.00)	0.292	-2.68 (-10.86, 2.21)	0.344	3.11 (-2.96, 12.78)	0.374	0.36 (-1.89, 3.13)	0.780	-2.93 (-11.73, 3.25)	0.358
	ADE	-0.04 (-0.75, 0.69)	0.906	-10.15 (-15.03, -4.86)	0.000*	-2.15 (-3.85, -0.65)	0.006*	-0.64 (-1.00, -0.27)	0.000*	0.02 (0.01, 0.04)	0.000*	-17.51 (-34.10, 0.03)	0.052	-9.79 (-36.75, 15.46)	0.462	0.16 (-9.92, 10.21)	0.978	-20.98 (-40.22, -1.47)	0.024*
	Total Effect	0.06 (-0.63, 0.79)	0.908	-10.29 (-15.02, -5.12)	0.000*	-1.95 (-3.67, -0.44)	0.010*	-0.60 (-0.95, -0.25)	0.000*	0.02 (0.01, 0.03)	0.002*	-20.18 (-38.09, -2.10)	0.026*	-6.68 (-33.05, 20.33)	0.610	0.52 (-9.45, 10.52)	0.896	-23.92 (-43.97, -4.39)	0.016*
	Prop. Mediated	0.04 (-4.57, 5.24)	0.884	0.01 (-0.12, 0.17)	0.800	-0.08 (-0.87, 0.10)	0.370	-0.04 (-0.38, 0.07)	0.434	-0.07 (-0.41, 0.07)	0.294	0.11 (-0.29, 0.68)	0.354	-0.06 (-4.31, 3.03)	0.796	0.01 (-2.84, 2.28)	0.952	0.11 (-0.21, 0.64)	0.350

CAD	ACME	0.00 (-0.32, 0.29)	0.982	-0.33 (-2.46, 1.25)	0.714	0.12 (-0.45, 0.92)	0.730	0.01 (-0.15, 0.14)	0.834	0.00 (-0.01, 0.00)	0.708	-1.05 (-8.59, 4.95)	0.748	4.24 (-12.50, 23.34)	0.588	-0.74 (-5.15, 2.45)	0.690	-0.88 (-8.61, 5.60)	0.808
	ADE	0.22 (-0.87, 1.22)	0.654	-10.31 (-15.38, -5.14)	0.000*	-2.57 (-4.66, -0.44)	0.010*	-0.74 (-1.21, -0.28)	0.002*	0.03 (0.01, 0.04)	0.004*	-16.01 (-36.34, 4.64)	0.128	-14.32 (-43.15, 14.39)	0.342	-3.05 (-13.92, 7.30)	0.584	-10.84 (-31.05, 9.26)	0.302
	Total Effect	0.22 (-0.92, 1.26)	0.646	-10.64 (-15.75, -5.33)	0.000*	-2.45 (-4.60, -0.29)	0.022*	-0.73 (-1.19, -0.25)	0.000*	0.02 (0.01, 0.04)	0.008*	-17.07 (-38.06, 2.90)	0.102	-10.08 (-43.26, 22.26)	0.542	-3.79 (-14.77, 6.95)	0.508	-11.72 (-33.14, 8.50)	0.258
	Prop. Mediated	0.01 (-2.42, 1.71)	0.940	0.02 (-0.18, 0.22)	0.714	-0.02 (-0.86, 0.25)	0.744	-0.01 (-0.30, 0.19)	0.834	-0.02 (-0.52, 0.15)	0.716	0.03 (-1.04, 0.98)	0.730	0.00 (-6.57, 4.66)	0.998	0.07 (-2.87, 2.95)	0.714	0.04 (-1.83, 1.59)	0.762
N_SPD	ACME	0.05 (-0.13, 0.30)	0.604	-1.11 (-3.63, 0.48)	0.232	0.23 (-0.16, 0.88)	0.302	0.02 (-0.08, 0.13)	0.672	0.00 (-0.01, 0.00)	0.480	-2.53 (-10.20, 2.02)	0.384	2.91 (-3.97, 13.30)	0.398	-0.40 (-3.86, 2.51)	0.790	-1.90 (-9.48, 3.51)	0.476
	ADE	-0.01 (-0.73, 0.75)	0.978	-9.54 (-15.06, -4.40)	0.000*	-2.24 (-3.87, -0.68)	0.008*	-0.63 (-0.99, -0.28)	0.000*	0.02 (0.01, 0.04)	0.000*	-17.68 (-37.04, 0.59)	0.060	-9.58 (-33.84, 16.08)	0.468	0.81 (-10.77, 12.15)	0.886	-23.06 (-44.96, -2.64)	0.028*
	Total Effect	0.05 (-0.67, 0.79)	0.912	-10.65 (-15.98, -5.32)	0.000*	-2.00 (-3.66, -0.43)	0.018*	-0.61 (-0.98, -0.26)	0.002*	0.02 (0.01, 0.04)	0.000*	-20.21 (-39.64, -1.78)	0.034*	-6.67 (-32.51, 20.02)	0.618	0.42 (-11.25, 11.84)	0.956	-24.97 (-47.15, -3.39)	0.024*
	Prop. Mediated	0.01 (-3.71, 3.41)	0.924	0.09 (-0.05, 0.34)	0.232	-0.08 (-0.88, 0.10)	0.312	-0.02 (-0.29, 0.14)	0.674	-0.05 (-0.42, 0.09)	0.480	0.10 (-0.23, 0.67)	0.398	-0.05 (-3.89, 4.24)	0.824	0.00 (-2.62, 2.52)	0.958	0.05 (-0.25, 0.48)	0.484
SPD	ACME	0.00 (-0.14, 0.14)	0.934	-0.03 (-1.03, 0.96)	0.896	0.02 (-0.28, 0.34)	0.894	0.00 (-0.07, 0.08)	0.886	0.00 (0.00, 0.00)	0.986	0.21 (-3.18, 4.00)	0.922	-0.11 (-5.14, 5.52)	0.952	-0.02 (-1.73, 1.55)	0.986	0.01 (-3.26, 3.29)	0.998
	ADE	0.00 (-0.67, 0.65)	0.994	-9.81 (-14.80, -5.23)	0.000*	-1.65 (-2.93, -0.22)	0.034*	-0.52 (-0.86, -0.20)	0.000*	0.02 (0.01, 0.03)	0.006*	-16.73 (-33.66, -0.07)	0.046*	-3.75 (-28.20, 20.54)	0.760	1.10 (-8.42, 10.42)	0.846	-21.57 (-40.88, -2.34)	0.028*
	Total Effect	0.00 (-0.66, 0.69)	0.992	-9.84 (-14.88, -4.90)	0.000*	-1.62 (-2.99, -0.17)	0.040*	-0.52 (-0.87, -0.18)	0.004*	0.02 (0.00, 0.03)	0.006*	-16.53 (-33.45, 0.38)	0.066	-3.86 (-28.03, 21.49)	0.778	1.08 (-8.71, 10.51)	0.838	-21.56 (-40.76, -2.35)	0.032*
	Prop. Mediated	0.01 (-1.33, 1.62)	0.894	0.00 (-0.12, 0.11)	0.896	0.00 (-0.41, 0.25)	0.902	0.00 (-0.20, 0.14)	0.890	0.00 (-0.20, 0.13)	0.988	0.00 (-0.56, 0.47)	0.956	0.01 (-1.47, 1.75)	0.890	0.01 (-1.04, 1.58)	0.904	0.00 (-0.28, 0.21)	0.974
N_SPM	ACME	0.14 (-0.25, 0.67)	0.544	-1.03 (-4.20, 1.72)	0.432	0.03 (-0.89, 1.00)	0.952	-0.01 (-0.22, 0.20)	0.882	0.00 (-0.01, 0.01)	0.990	-3.23 (-13.58, 6.29)	0.504	4.90 (-9.82, 20.21)	0.518	-1.08 (-5.68, 2.92)	0.574	-0.55 (-13.44, 10.78)	0.968
	ADE	-0.19	0.662	-8.69	0.000*	-1.90	0.038*	-0.57	0.002*	0.02	0.012*	-12.22	0.192	-6.83	0.632	-0.99 (-9.41, 7.32)	0.798	-17.93	0.112

		(-1.01, 0.55)		(-14.14, -3.45)		(-3.70, -0.18)		(-0.93, -0.20)		(0.00, 0.04)		(-32.28, 7.11)		(-33.17, 19.09)				(-38.48, 4.64)	
	Total Effect	-0.05 (-0.82, 0.66)	0.892	-9.71 (-14.62, -5.28)	0.000 *	-1.87 (-3.43, -0.36)	0.016 *	-0.58 (-0.89, -0.25)	0.000 *	0.02 (0.01, 0.03)	0.008 *	-15.45 (-33.06, 1.90)	0.084	-1.93 (-27.12, 21.73)	0.870	-2.07 (-9.71, 5.20)	0.566	-18.48 (-36.60, -0.03)	0.050
	Prop. Mediated	0.00 (-7.57, 8.83)	0.996	0.10 (-0.19, 0.45)	0.432	-0.02 (-0.84, 0.76)	0.952	0.02 (-0.43, 0.45)	0.882	0.00 (-0.61, 0.53)	0.994	0.19 (-1.10, 2.34)	0.544	-0.08 (-8.49, 7.68)	0.928	0.10 (-5.68, 8.34)	0.860	0.01 (-1.21, 1.53)	0.986
SPM	ACME	-0.11 (-0.55, 0.26)	0.490	0.43 (-2.61, 3.62)	0.742	-0.05 (-1.07, 0.83)	0.862	-0.02 (-0.24, 0.20)	0.822	0.00 (-0.01, 0.01)	0.822	-2.29 (-11.49, 4.75)	0.516	-0.17 (-14.36, 15.83)	0.962	-1.94 (-8.22, 1.67)	0.336	3.16 (-6.74, 16.20)	0.552
	ADE	0.24 (-0.57, 1.02)	0.538	-10.78 (-17.36, -4.08)	0.004 *	-1.85 (-3.90, 0.05)	0.058	-0.56 (-1.02, -0.07)	0.022 *	0.03 (0.01, 0.04)	0.012 *	-17.71 (-35.72, 0.72)	0.064	-4.92 (-40.21, 29.37)	0.784	0.95 (-9.26, 11.46)	0.858	-30.61 (-59.83, -3.74)	0.024 *
	Total Effect	0.13 (-0.63, 0.89)	0.710	-10.35 (-16.54, -4.11)	0.002 *	-1.90 (-3.67, -0.02)	0.048 *	-0.58 (-0.98, -0.15)	0.008 *	0.02 (0.01, 0.04)	0.008 *	-20.00 (-38.90, -2.42)	0.028 *	-5.08 (-40.78, 29.33)	0.752	-1.00 (-11.07, 9.43)	0.828	-27.45 (-56.05, -0.99)	0.042 *
	Prop. Mediated	-0.06 (-7.41, 6.29)	0.880	-0.03 (-0.50, 0.26)	0.744	0.02 (-0.75, 0.92)	0.862	0.02 (-0.46, 0.61)	0.826	-0.02 (-0.63, 0.40)	0.826	0.08 (-0.39, 0.83)	0.536	0.01 (-4.14, 4.73)	0.978	0.08 (-4.31, 6.76)	0.856	-0.08 (-1.90, 0.43)	0.566

Data represent estimates from exploratory indirect-effect analyses using the R mediation package with nonparametric bootstrap resampling (1,000 iterations). ACME, ADE, and total effect estimates are presented with 95% confidence intervals (CI). * Nominal $p < 0.05$. p values are nominal and were not adjusted for multiplicity. ACME and ADE terminology follows the output of the R mediation package and should be interpreted as exploratory indirect-effect estimates rather than evidence of causal mediation. Analyses were adjusted for age and baseline BMI. Abbreviations: BMI, body mass index; WBC, white blood cell; HOMA-IR, homeostasis model assessment of insulin resistance; QUICKI, quantitative insulin sensitivity check index; HDL, high-density lipoprotein; LDL, low-density lipoprotein; N_PUT, N-acetylputrescine; N_CAD, N-acetylcadaverine; DAP, 1,3-diaminopropane; PUT, putrescine; CAD, cadaverine; N_SPD, N-acetylspermidine; SPD, spermidine; N_SPM, N-acetylspermine; SPM, spermine; ACME, average causal mediation effect; ADE, average direct effect; CI, confidence interval.
